# Peripheral immune cell dysregulation following diffuse traumatic brain injury in pigs

**DOI:** 10.1186/s12974-024-03317-y

**Published:** 2024-12-18

**Authors:** Kathryn L. Wofford, Kevin D. Browne, David J. Loane, David F. Meaney, D. Kacy Cullen

**Affiliations:** 1 Center for Brain Injury & Repair, Department of Neurosurgery, University of Pennsylvania, 105 Hayden Hall, 3320 Smith Walk, Philadelphia, PA 19104 USA; 2 Center for Neurotrauma, Neurodegeneration and Restoration, Corporal Michael J. Crescenz VA Medical Center, Philadelphia, PA 19104 USA; 3 School of Biochemistry and Immunology, Trinity Biomedical Sciences Institute, Trinity College, Dublin, Ireland; 4 Department of Bioengineering, University of Pennsylvania, Philadelphia, PA 19104 USA

**Keywords:** Traumatic brain injury, Peripheral blood mononuclear cells, Closed-head diffuse traumatic brain injury, Peripheral immune system, Immunosuppression, Immunodysregulation, Pig

## Abstract

Traumatic brain injury (TBI) is a global health problem affecting millions of individuals annually, potentially resulting in persistent neuropathology, chronic neurological deficits, and death. However, TBI not only affects neural tissue, but also affects the peripheral immune system’s homeostasis and physiology. TBI disrupts the balanced signaling between the brain and the peripheral organs, resulting in immunodysregulation and increasing infection susceptibility. Indeed, secondary infections following TBI worsen neurological outcomes and are a major source of mortality and morbidity. Despite the compelling link between the damaged brain and peripheral immune functionality, little is known about how injury severity affects the peripheral immune system in closed-head diffuse TBI, the most common clinical presentation including all concussions. Therefore, we characterized peripheral blood mononuclear cells (PBMCs) and plasma changes over time and across injury severity using an established large-animal TBI model of closed-head, non-impact diffuse rotational acceleration in pigs. Across all timepoints and injury levels, we did not detect any changes to plasma cytokine concentrations. However, changes to the PBMCs were detectable and much more robust. We observed the concentration and physiology of circulating PBMCs changed in an injury severity-dependent manner, with most cellular changes occurring within the first 10 days following a high rotational velocity injury. Here, we report changes in the concentrations of myeloid and T cells, changes in PBMC composition, and changes in phagocytic clearance over time. Together, these data suggest that following a diffuse brain injury in a clinically relevant large-animal TBI model, the immune system exhibits perturbations that are detectable into the subacute timeframe. These findings invite future investigations into therapeutic interventions targeting peripheral immunity and the potential for peripheral blood cellular characterization as a diagnostic tool.

**Supplementary Information:**

The online version contains supplementary material available at 10.1186/s12974-024-03317-y.

## Paper highlights

- We sought to characterize changes to the peripheral immune system following a clinically relevant model of diffuse closed-head rotational TBI in pigs.
- The cellular composition and physiology of the immune system is changed following a closed-head rotational brain injury in pigs in an injury severity-dependent manner.
- Innate and adaptive cellular changes were detectable and robust within these study parameters, however, changes to plasma cytokines were not detectable.
- Many peripheral immune cellular changes persisted into subacute timepoints.
- Together, these data suggest that following a diffuse brain injury in a clinically relevant large-animal model, the immune system exhibits perturbations that are detectable into the subacute timeframe, offering opportunities for peripheral cellular temporal alterations to be a diagnostic tool and potential targets for therapeutic intervention.

**Supplementary Information:**

The online version contains supplementary material available at 10.1186/s12974-024-03317-y.

## Introduction

Traumatic brain injury (TBI) is a global health problem, affecting approximately 69 million people every year, potentially resulting in disrupted neuronal circuitry, persistent neurological deficits, increased susceptibility to secondary infections, and death [1–5]. Mechanical damage to the brain during TBI not only disrupts neurological tissue but also affects the peripheral immune system’s homeostasis and physiology. Under healthy conditions, the brain and immune system continuously signal to one another, generating a protective and balanced environment that minimizes the risks of infection and encourages endogenous immune-mediated tissue regeneration [6, 7]. However, when the brain is injured during trauma, the balanced crosstalk between the two organ systems becomes disrupted, dysregulating signals to and within the immune system [2, 3, 8]. For example, damage to the hypothalamus, pituitary, or caudal nucleus of the solitary tract (cNST) disrupts neuronal circuitry that regulates immune system functionality through the hypothalamic-pituitary-adrenal axis, the sympathetic-adrenal-medullary axis, and the parasympathetic nervous system [2, 6]. TBI-induced damage, especially to these different regulatory neuronal circuits, can result in immunodysregulation, facilitating hyperactive or depressed immune responses, leaving the body susceptible to infection and subsequent damage [9, 10]. Furthermore, the immune system responds to damage-associated molecular patterns (DAMPs) released from the injured brain into the circulation [11]. Circulating peripheral immune cells react to signals of trauma by increasing their concentration in whole blood (via increasing proliferating rates or exiting reserve tissues) or by decreasing their concentration in whole blood (via homing to damaged tissues or dampening proliferation rates).

In clinical cases of severe TBI, secondary infection is a common complication and a leading cause of morbidity and mortality [2, 8, 12, 13]. Furthermore, a dysregulated immune system also affects the brain and its recovery potential. Indeed, subjects that experience an infection after TBI have an increased likelihood of delayed recovery and poorer neurological outcomes [14, 15]. In addition to these acute changes, disruption of immune homeostasis can increase the risk for long-term morbidity and is linked to neurological degeneration and dementia [16, 17].

The vast majority of TBI cases are closed-head diffuse injuries generated by rapid rotational acceleration, typically occurring during falls, traffic collisions, or assaults [4, 18, 19]. Closed-head diffuse TBI frequently generates more subtle, diffuse pathology that is not overtly evident with clinical imaging or gross pathological characterization [20, 21]. However, closed-head diffuse TBI results in persistent neural and glial changes that are detectable chronically after trauma [17, 22, 23]. To model this most common type of clinical TBI presentation, we employed an established, closed-head, non-impact diffuse rotational velocity TBI in pigs [24–28]. This injury model exhibits neuronal pathology in the hypothalamus region and brainstem, areas that contain immunomodulatory neuronal circuitry [29, 30]. We sought to determine if immunodysregulation was detectable and mirrored clinical trends across injury severities within this preclinical model. Even though our model of closed-head diffuse rotational velocity TBI generates a milder injury relative to the severe clinical TBIs previously associated with immunodysregulation, we hypothesized that closed-head diffuse TBI could induce detectable immunodysregulation in an injury severity-dependent manner. To test this hypothesis, we collected peripheral whole blood from study subjects and surveyed changes to the peripheral blood mononuclear cells (PBMCs) and plasma over time.

## Methods

### Animal enrollment and handling

All animals utilized within these studies were kept and managed in accordance with the Guide for the Care and Use of Laboratory Animals [31] and followed ARRIVE (Animal Research: Reporting of In Vivo Experiments) Guidelines. Protocols were approved by the University of Pennsylvania’s Institutional Animal Care and Use Committee. Sexually mature, young adult (6-month-old) female Yucatan minipigs [32, 33], purchased from Sinclair Research, with an average weight of 31.2 kg were utilized. Upon arrival, animals were quarantined to confirm an absence of transmissible zoonotic pathogens and to allow acclimation. Animals were housed indoors in a facility accredited by the Association for Assessment and Accreditation of Laboratory Animal Care International with food and water available *ad libitum*.

Animals were randomly assigned to a sham procedure (*n* = 3), to a moderate rotational velocity injury procedure (*n* = 4), or to a high rotational velocity injury procedure (*n* = 7). Animals experiencing a high rotational velocity injury procedure were survived for 3 (*n* = 3) or 14 (*n* = 4) days post-injury (dpi). All subjects in the sham and moderate rotational velocity injury groups survived 14 days post-injury. Animals were fasted overnight prior to procedure with water remaining *ad libitum*.

### Central line surgical procedure

Animals were induced with a cocktail of ketamine (20–30 mg/kg) and (0.4–0.6 mg/kg) midazolam, administered intramuscularly. Animals were intubated with an endotracheal tube (5.5 mm) and a plane of anesthesia was maintained with 1–5% isoflurane per 2–3 L of 100% O_2_ for the duration of the procedure. Within these ranges, anesthetic was titrated to maintain desired depth of anesthesia and physiological safe heart rate, respiratory rate, and arterial oxygen saturation (heart rate between 100 and 130 beats per minute, respirations between 9 and 12 breaths per minute, and SpO_2_ between 97 and 100%). Animals were given 0.01 mg/kg glycopyrrolate subcutaneously and eye lubricant was applied. Normothermia was maintained with a forced-air temperature management system and blankets.

An indwelling catheter was placed in all animals prior to sham or brain injury procedure. Briefly, the sternal and dorsal surgical sites were shaved and aseptically cleaned. Subjects were positioned in a dorsal recumbency and draped. Bupivicaine (1 mg/kg) was injected subcutaneously, and an incision was made lateral to midline in the jugular furrow. Tissue was blunt dissected away to expose the cephalic vein. The vein was catheterized and secured in place with silk sutures. Then, the catheter was tunneled, via trocar, through the subcutaneous space, exiting the skin near the dorsal aspect of the scapula. Excede (5 mg/kg) was administered intramuscularly at the time of the surgery as a prophylactic against bacterial infection. Incision sites were sutured and sealed with sterile tape. The catheter was maintained with daily flushes of saline and was protected in a fitted vest to prevent animal interference. Throughout the study, there were no signs of swelling, redness, or sensitivity at surgical sites.

### Closed-head diffuse brain injury

**Fig. 1:**
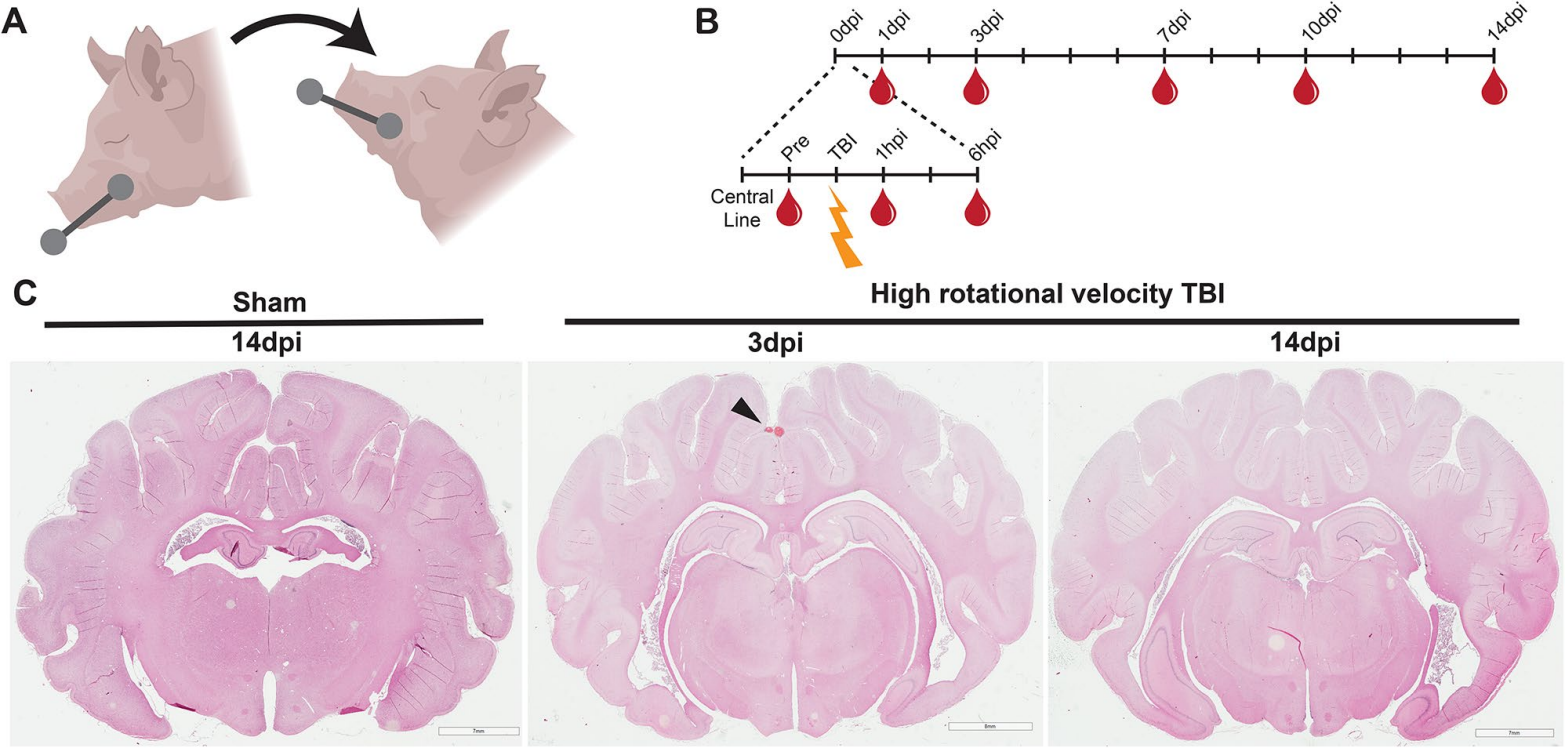
Experimental procedure, sample collection timeline, and gross pathology of closed head rotational TBI. Animal subjects experienced a sham or a closed-head rotational acceleration injury in the sagittal plane (**A**). Whole blood was collected prior to injury and up to 14 days post-injury as a repeated measure (**B**). H&E staining of coronal brain sections after a sham or a high rotational velocity TBI exemplify modest changes to the brain 3dpi – such as hemorrhage in midline regions (arrowhead) – but not 14 dpi (**C**)

To induce a closed-head rotational acceleration diffuse brain injury in the injury cohorts, anesthetized animals were mounted to a padded bite plate that was connected to a HYGE pneumatic actuator. The mouth was carefully positioned and secured to the padded bite plate with adjustable snout cables. The HYGE device generates purely impulsive, non-impact rotational movement. This generates rotational forces scalable to clinical TBIs [26, 34, 35]. In this study, subjects experienced rotation in the sagittal plane (in plane with the brain stem) at two levels: a moderate rotational velocity injury (average 90.7 radians per second (rad/s)) or a high rotational velocity injury (average 109.5 rad/s; Fig. 1A; Table 1). Angular displacement over time was recorded in LabVIEW with two magneto-hydrodynamic sensors (Applied Technology Associates, Albuquerque, NM; sampling rate at 10 kHz). Sham animals received all procedures except head rotation.

**Table 1:** Average injury kinematics and recovery times (mean ± standard deviation)

	Sham	Moderate Rotational Velocity TBI	High Rotational Velocity TBI
Body weight (kg)	30.5 ± 2.0	31.8 ± 1.3	31.3 ± 4.1
Maximum angular velocity (rad/s)	N/A	90.7 ± 3.3	109.5 ± 2.3
Maximum angular acceleration (rad/s^2^)	N/A	14,995.5 ± 2,028.3	22,571.3 ± 1,895.8
Minimum angular acceleration (rad/s^2^)	N/A	-21,979.3 ± 2,977.8	-32,688.4 ± 1,821.4
Recovery duration (min)	26 ± 14	29 ± 23	65 ± 24

Following injury, animals were removed from the bite plate, examined for oral or dental injuries, and returned to their housing units. Isoflurane was turned off and buprenorphine sustained release (0.1 mg/kg) was administered subcutaneously. Extubation occurred and was recorded when prompted, as determined by chewing, swallowing, or coughing. Standing time was recorded in order to calculate the recovery duration for each animal, defined as the difference between extubation and standing times. Animals were continuously monitored until ambulatory and stable and then at least daily thereafter to ensure health and stability. Altogether, this injury procedure generates brain injury but may also induce modest oral injuries (such as bruising to the snout and soft tissue) in most animals. Furthermore, placement of the central line is also an injurious procedure that places a foreign object in the body. While multiple tissues outside of the brain may be modestly disrupted during these procedures, this is not generally considered a model of polytrauma, which may be better represented by multiple severe injuries to several tissues that increase risk of morbidity and mortality [36].

### Blood collection and post processing

Whole blood was collected prior to injury and at designated timepoints following the injury procedure (Fig. 1B). To avoid effects of circadian flux, samples were collected at the same time during the day (ZT2; +/- 1 h), with the exception of the 1 and 6 h post-injury (hpi) timepoints. The total volume of blood taken over the course of the whole study never exceeded 1% of the animal’s weight. The central line was flushed daily with at least 10 mL of saline to maintain catheter patency. After flushing the line with saline, the line was primed, and 10–20 mL of whole blood was collected in EDTA-coated tubes. Thereafter, lines were flushed again with saline to prevent clotting inside the catheter lumen.

To isolate white blood cells and plasma, whole blood samples were diluted 1:1 in sterile 2mM ethylenediaminetetraacetic acid (EDTA) and gently layered on top of room temperature Ficoll solution. Samples were centrifuged at 400xG for 20 min at room temperature with no breaking to separate out peripheral blood mononuclear cells (PBMCs) and plasma from red blood cells and granulocytes. Plasma was collected, aliquoted, and stored at -80˚C. PBMCs were incubated with 1X red blood cell lysis buffer, washed three times, and counted. PBMCs were resuspended in freezing media (50% RPMI, 40% heat inactivated FBS, 10% DMSO) and stored at -80˚C before being transferred to the vapor phase of liquid nitrogen for longer term storage.

### Terminal procedure and tissue collection

At the designated timepoint, animals were weighed, induced, and intubated as described above. Subjects were transcardially perfused with 0.9% heparinized saline. When the blood ran clear, the spleen and left thymus were collected, submerged in DPBS with 1% Pen/Strep, and kept on ice. Thereafter, transcardial perfusion was continued with 10% neutral buffered formalin. The remaining perfusion-fixed thymus tissue was extracted and post-fixed for one week at 4˚C. Decapitated tissue was post-fixed in 10% formalin for 24 h. The brain was extracted and post-fixed for one week at 4˚C.

Non-fixed spleen and thymus tissue was weighed and imaged. Spleen tissue was partitioned for fresh cell flow cytometry and fixed histological analyses. Non-fixed spleen and thymus tissues were morselized, incubated with RBC lysis buffer for 10 min, and cell suspensions were passed through a 70 μm filter. Splenocytes were reincubated with RBC lysis buffer a second time. Splenocytes and thymocytes were washed twice in RPMI 1640 without phenol red, counted, and frozen.

### Flow cytometry

To complete immunophenotyping, PBMCs, splenocytes, and thymocytes were thawed, counted, and blocked with a cocktail of CD16 (1:200; MEM-168; Invitrogen) and CD32 (1:140; AT10; Invitrogen). To exclude dead cells, samples were incubated with Live/Dead fixable yellow (ThermoFisher) according to manufacturer’s instructions. Samples were stained with primary antibodies CD52-FITC (11/305/44); CD172a-PE (74-22-15 A); CD8a-AF647 (76-2-11); CD3e-PerCP-Cy5.5 (BB23-8E6-8C8); and CD4a-PE-Cy7 (74-12-4) purchased from BD Biosciences. Stained cells were fixed with 5% neutral buffered formalin.

To characterize reactive oxygen species (ROS) production, cells were incubated with phorbol 12-myristate 13-acetate (PMA; 27.5 µg/mL) for 30 min at 37˚C and then incubated with dihydrorhodamine (DHR) 123 (Millipore Sigma; D1054) for 20 min at 37˚C before blocking. Cells in the ROS assay were stained with Live/Dead stain and the antibodies above, except CD52 was excluded due to emission overlap with the DHR 123. Following staining, cells were fixed. To characterize phagocytic capacity, cells were incubated with PMA (27.5 µg/mL) for 30 min at 37˚C and then incubated with one of three phagocytic targets: *E. coli* particles (Invitrogen; E2861), *S. aureus* particles (Invitrogen; S2851), or polystyrene particles (Millipore Sigma; L4655) for 30 min at 37˚C. Phagocytic targets were incubated with cells at a ratio of 10 targets per cell. Cells in the phagocytic assay were stained with Live/Dead stain and fixed.

Data were acquired on an LSRII using FACsDiva and analyzed with FlowJo (v10.9.0). All experiments were completed with positive, negative, dead cell, and fluorescence minus one (FMO) controls. Positive and negative controls were utilized to calculate fluorescent compensation. FMO controls were utilized to gate positive and negative signal in experimental samples (Supp. Figure 1).

### Plasma cytokine assay

Blood plasma samples were characterized for a panel of ten cytokines using a previously validated multiplexed sandwich ELISA-based quantitative array (Raybiotech; Quantibody). The quantitative protein array detects ten pro- and anti-inflammatory cytokines including interleukin (IL)-1β, IL-4, IL-6, IL-8, IL-10, IL-12, granulocyte-macrophage colony-stimulating factor (GM-CSF), interferon (IFN)-γ, transforming growth factor (TGF)-β1, and tumor necrosis factor (TNF)-α. The assay was completed according to the manufacturer’s instructions. Slides were scanned by the manufacturer. Calculated concentrations were multiplied by the EDTA dilution factor. Data are presented as the calculated circulating concentration.

### Peripheral tissue histology

Fixed spleen and thymus tissue were sectioned and stained for hematoxylin and eosin (H&E) by the University of Pennsylvania Veterinary Comparative Pathology Core. A trained and blinded pathologist analyzed the tissue.

### Brain tissue histology and imaging

Beginning at the optic chiasm, brains were blocked into 5 mm thick coronal blocks. Brain blocks were processed, paraffin embedded, and 8 μm sections were collected with rotary microtome. Three sections from each subject were utilized to ensure observations were representative trends across the whole brain: one containing rostral thalamus tissue (approximately 1 mm posterior to the optic chiasm); one containing the caudal hippocampus (approximately 10 mm posterior to the optic chiasm); and one containing the occipital/brainstem (approximately 15 mm posterior to the optic chiasm). H&E sections were imaged at 20X optical zoom using an Aperio CS2 digital slide scanner (Leica Biosystems Inc., Buffalo Grove, IL). Fibrinogen (Abcam, #183109; 1:5,000) staining was completed with DAB (Vector Laboratories; #SK-4100) with hematoxylin counterstain across three anatomical sections for all subjects. A blinded technician selected fibrinogen^+^ regions and quantified their area. Fibrinogen burden was defined as the summed area across three brain sections for each animal.

### Statistical analyses

All data were statistically assessed with the appropriate test. Following flow cytometry compensation, the geometric mean was utilized to quantify fluorescence intensity within defined populations and gates. Yucatan minipigs are non-syngenic animals that can exhibit high animal-to-animal variability. To reduce between-subjects variability, dependent measures were occasionally subtracted from animal-matched pre-injury levels to determine changes relative to baseline and presented as supplemental information (Supp. Figure 3). Changes in immune cell populations and behaviors over time (both normalize and non-normalized) were analyzed with mixed-effects models. Blood and plasma measures over time from animal replicates were treated as repeated measures. Differences between injury conditions, such as changes in relative spleen mass or fibrinogen burden, were assessed with one-way ANOVAs. Significance was determined with Tukey’s correction for multiple comparisons. All data is represented with the mean and standard deviation. Analyses were completed in GraphPad Prism version 10.3.1 (464).

## Results

### Closed-head rotational acceleration induces brain injury

Closed-head rotational acceleration TBI was generated in anesthetized pigs by imparting a purely impulsive, non-impact rotation in the sagittal plane (Fig. 1A). Injured animals were subjected to a moderate rotational velocity injury with an average peak angular velocity of 90.7 rad/s and average peak angular acceleration of 14,995.5 rad/s^2^ or to a high rotational velocity injury with an average peak angular velocity of 109.5 rad/s and average peak angular acceleration of 22,571.3 rad/s^2^ (Table 1).

**Fig. 2:**
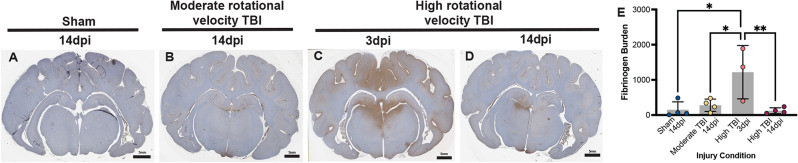
The blood protein, fibrinogen (brown), is negligible following a sham injury (**A**) and 14 days after a moderate rotational velocity TBI (**B**). Fibrinogen staining is extensive 3 days after a high rotational velocity TBI in midline brain regions (**C**) but is largely diminished 14 dpi (**D**). Fibrinogen burden across brain sections and across injury conditions (**E**)

Rapid rotational acceleration in the sagittal plane at these injury speeds generates subtle gross pathological observations but exhibits cellular pathology including neuronal mechanoporation, microglia morphological changes, and mitochondrial stress acutely following injury [26, 37–39]. Consistent with these reports, subjects experiencing a moderate rotational velocity TBI exhibited no macroscopic pathological changes (Supp. Figure 2). High rotational velocity TBI generated occasional microhemorrhage in the midline or periventricular space 3 days post-injury (dpi; Fig. 1C, arrowhead). Neither injury condition exhibited cavitation, major hemorrhage, or lesions, which is more common in other injury models [24, 40, 41]. However, blood-brain barrier (BBB) integrity was diminished three days after a high rotational velocity TBI, allowing the blood protein fibrinogen to extravasate into the brain parenchyma (Fig. 2). Overall, this large-animal injury model at the moderate rotational velocity generated pathology mimetic of mild clinical brain injury while the high rotational velocity generated pathology mimetic of complex mild to moderate clinical brain injury [4].

All animals recovered following the sham or injury procedure, but injured animals exhibited a modest extension in recovery time, defined as the duration between extubation and standing times [35]. Moderate rotational velocity TBI slightly extended the average recovery period from 26 min to 29 min, while a high rotational velocity TBI further extended the average recovery time to 65 min (Table 1; *p* = 0.07 relative to sham).

### PBMC numbers and composition changes following closed-head brain injury

**Fig. 3:**
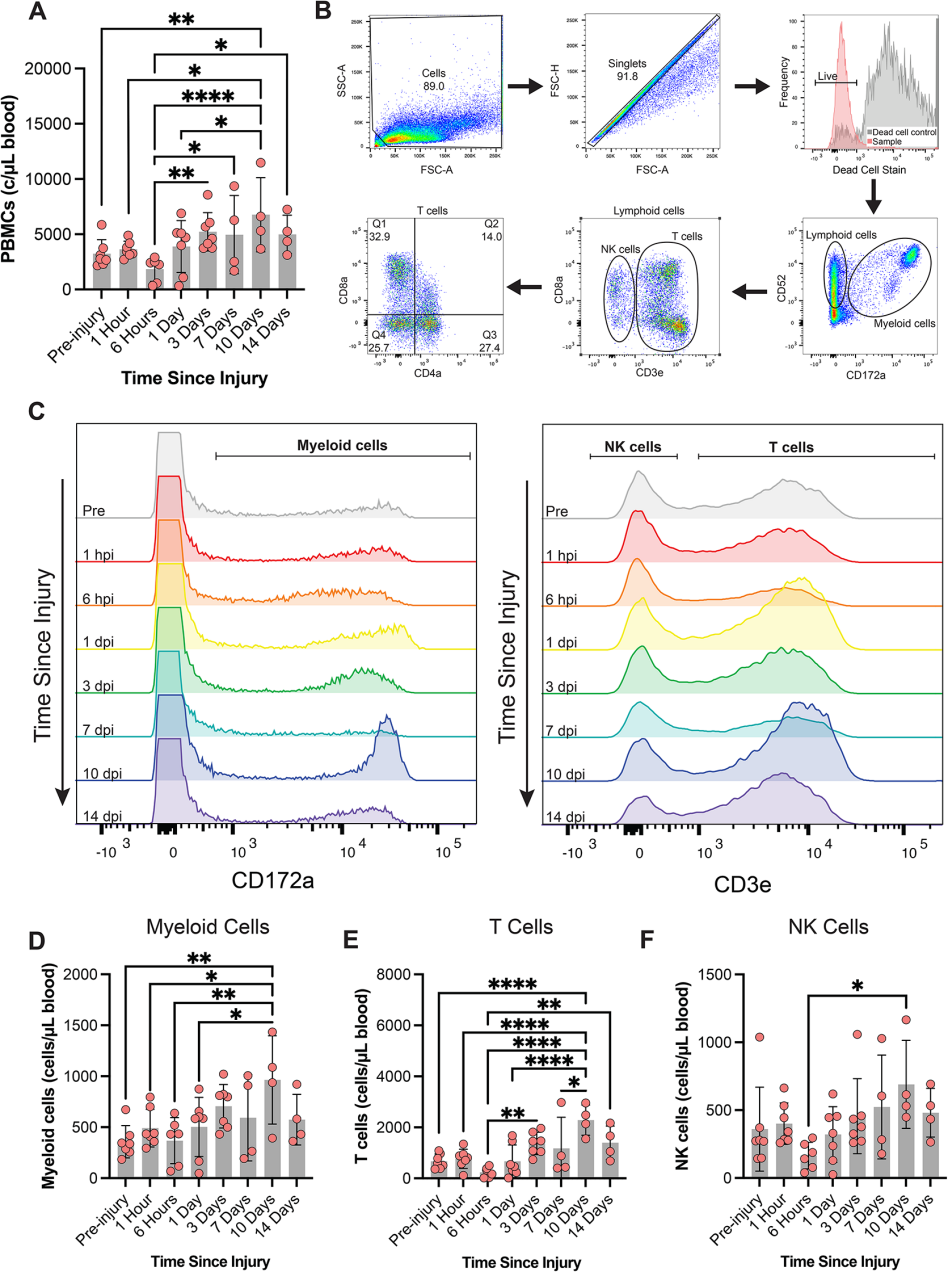
Changes to circulating peripheral immune cell populations over time after a TBI. The concentration of circulating PBMCs in whole blood is upregulated 10 days after a high rotational velocity TBI (**A**). Flow cytometry staining and gating methodology from a representative animal (**B**, **C**). Population dynamics of myeloid cells (**D**), T cells (**E**), and NK cells (**F**) over time after a high rotational velocity TBI

Our primary interest was to determine how closed-head brain injury affects peripheral immune system homeostasis. Circulating PBMCs are sentinels of the body, sensing and responding to infection, damage, and disease. Thus, we first characterized the concentration of PBMCs in circulation to assess if there were any alterations to the peripheral immune system. PBMCs from whole blood were collected as repeated measures prior to injury, and up to 14 days post injury (Fig. 1B). While we did not observe changes to circulating PBMC concentration following a moderate rotational velocity TBI (Supp. Figure 2), we did observe changes following a more injurious, high rotational velocity TBI. The concentration of circulating PBMCs exhibited a non-significant decrease 6 hpi. Thereafter, PBMC concentrations increased at later timepoints with PBMCs significantly increasing in concentration 10 dpi relative to pre-injury levels (Fig. 3A).

PBMCs are a heterogenous population comprised of several subtypes of white blood cells. Using established staining and gating methodologies [42], we characterized myeloid and lymphoid cell populations (Fig. 3B). In PBMCs extracted following a high rotational velocity TBI, the number of circulating myeloid cells increased 10 dpi relative to pre-injury concentrations (Fig. 3C-D).

**Fig. 4:**
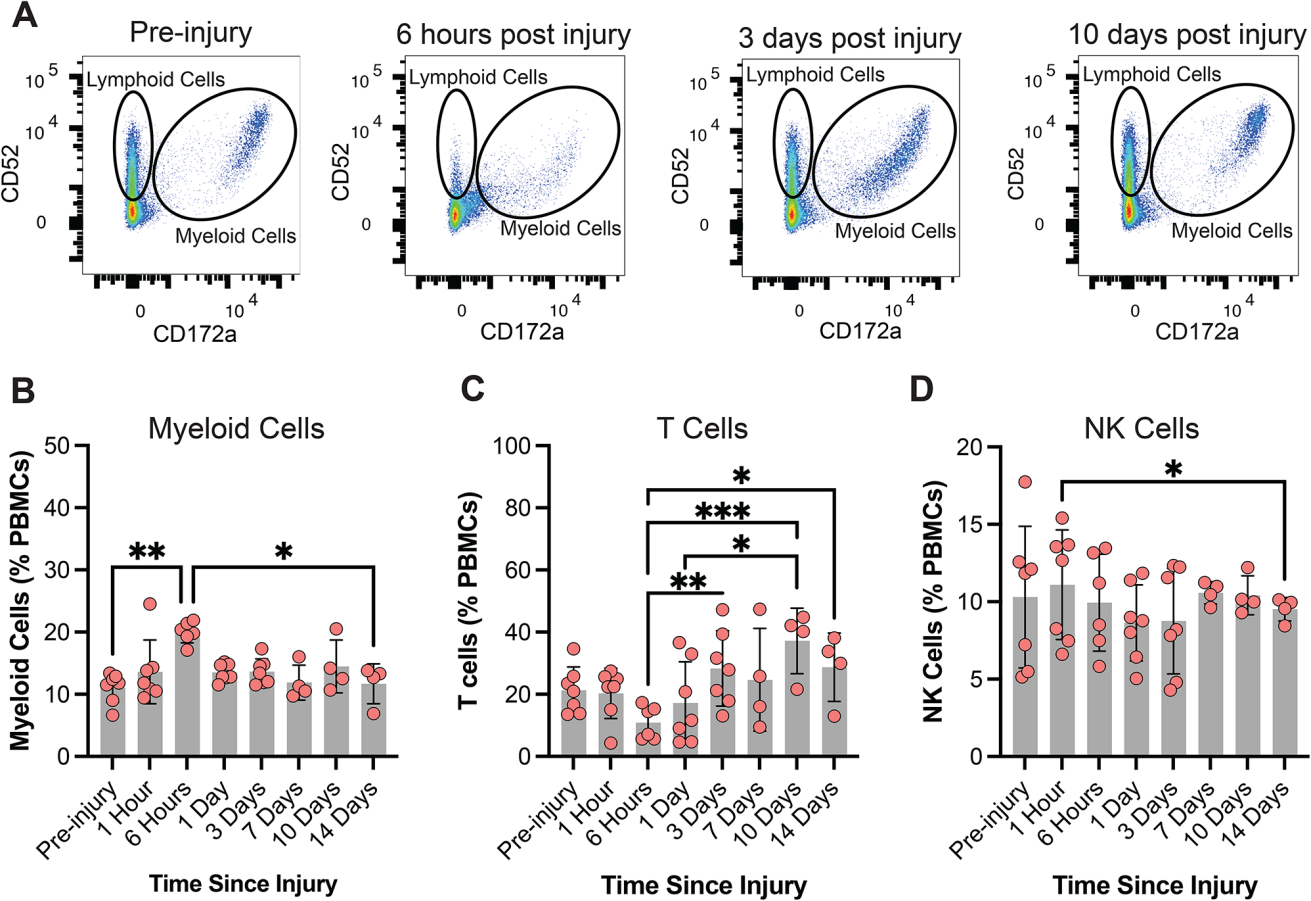
PBMC composition over time after TBI. Population gating strategy of PBMCs (**A**) detects transient and subacute changes to myeloid cells (**B**) increases in T cells (**C**), and subtle changes to NK cells (**D**)

The concentration of circulating T cells exhibited a non-significant decline at acute timepoints and then increased relative to acute concentrations (Fig. 3E). The number of circulating T cells increased 10 dpi relative to pre-injury concentrations (Fig. 3E). The concentration of circulating NK cells was increased 10 dpi relative to the 6 hpi timepoint (Fig. 3F). Moderate rotational velocity TBI generated modest changes in myeloid cell, T cell, or NK cell concentrations over time, and the sham procedure resulted in no significant differences over time (Supp. Figure 3). Together, these changing immune cell subtypes altered the relative proportion of circulating immune cell subtypes following a high rotational velocity TBI. The percentage of PBMCs that were myeloid cells significantly increased 6 hpi and then returned to baseline levels for subsequent timepoints (Fig. 4A-B). The percentage of T cells increased 3, 10, and 14 dpi relative to the 6 hpi timepoint (Fig. 4C). The percentage of NK cells decreased 14 dpi relative to the 1 hpi timepoints (Fig. 4D).

**Fig. 5:**
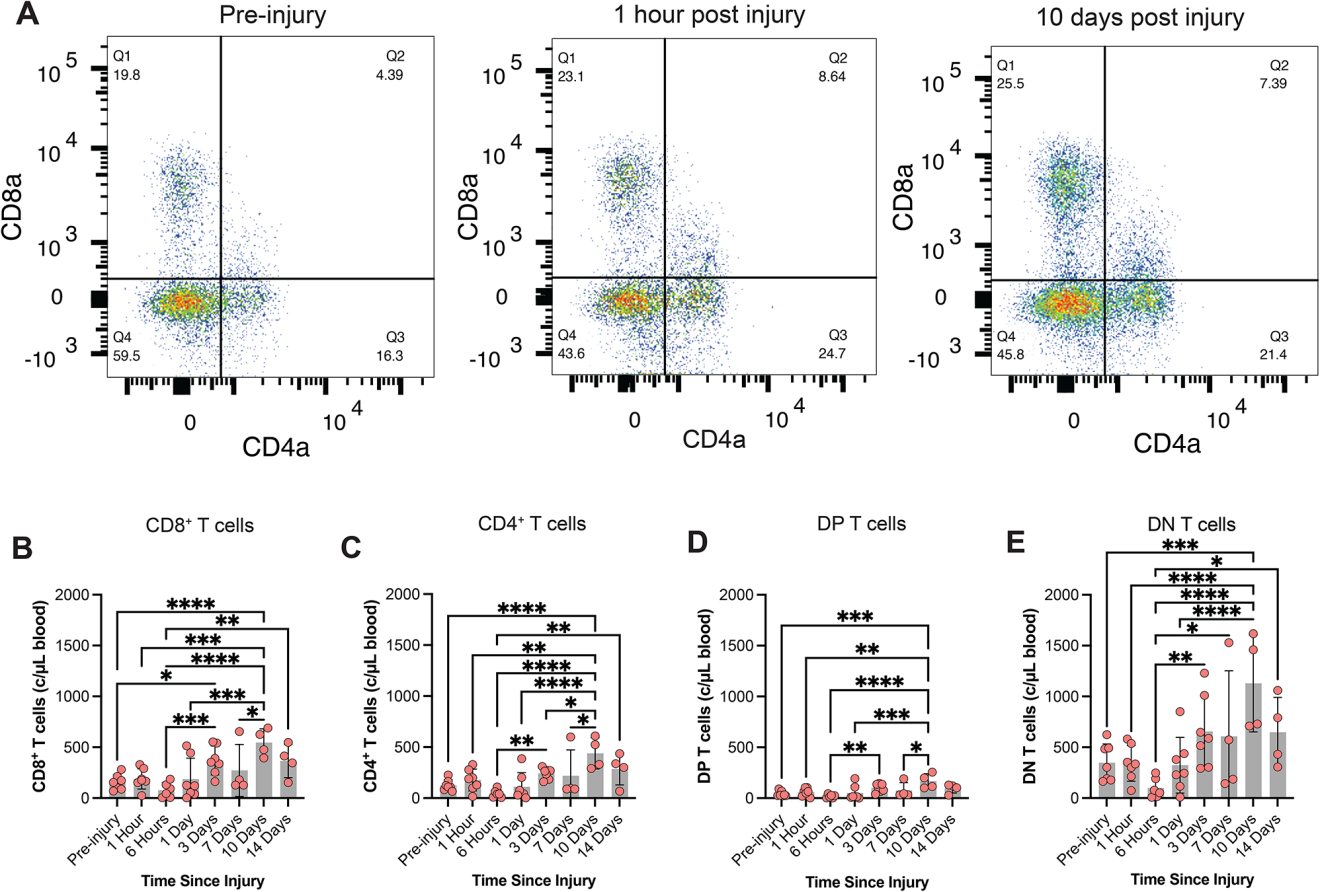
T cell subtype changes over time after a high rotational velocity TBI. T cell subset dynamics (**A**) including CD8^+^ (**B**), CD4^+^ (**C**), double positive (DP; **D**), and double negative (DN; **E**) T cell subset dynamics over time after a high rotational velocity TBI

T cells can drive both immunogenic and immuno-regulatory functions [43]. To investigate which T cell subsets were driving changes in the T cell population size, we analyzed T cells according to CD4 and CD8 expression. Like humans, pig T cells can be categorized as CD4 + T helper cells or CD8 + cytotoxic T cells [42]. Unlike humans, pigs have a large population of double negative (CD4-/CD8-) T cells that encompass γδ T cells and null T cells [42]. All T cell subsets exhibited a non-significant decrease in concentration at acute timepoints and an increase in concentration at later timepoints (Fig. 5A-E). All T cells subsets were significantly upregulated 10 dpi relative to pre-injury levels. CD8 + cytotoxic T cells were also significantly upregulated 3 dpi relative to pre-injury levels (Fig. 5B). Subjects receiving a moderate rotational velocity TBI exhibited very modest shifts in T cell subtypes, with increasing double positive T cells 3 dpi relative to the 1 hpi timepoint (Supp. Figure 4). Within T cells, the percentage of CD4 + T helper cells decreased 1 dpi, relative to the 6 hpi timepoint following a high rotational velocity TBI (Supp. Figure 5). The percentage of T cells that were CD8 + remained unchanged over time after a high rotational velocity TBI but increased 6 hpi after a moderate rotational velocity TBI (Supp. Figure 5). The ratio of CD8 to CD4 T cells remained unchanged across all timepoints and injury conditions (Supp. Figure 6).

### Immune cell physiology is altered following TBI

Immune cell population shifts indicate that PBMCs may respond to cues from the injured brain. Therefore, we wanted to examine if immune cell reactivity and function was altered in response to a challenge following TBI. Respiratory burst and the production of reactive oxygen species (ROS) is a major function that immune cells utilize to neutralize pathogens and signal to one another [44]. PMA-stimulated PBMCs extracted at different times after a moderate or high rotational velocity TBI did not change their relative ROS-production relative to pre-injury levels (Supp. Figure 7). Stimulated immune cells extracted 3 days after a sham procedure reduced their ROS production relative to 1 dpi cells (Supp. Figure 7).

**Fig. 6:**
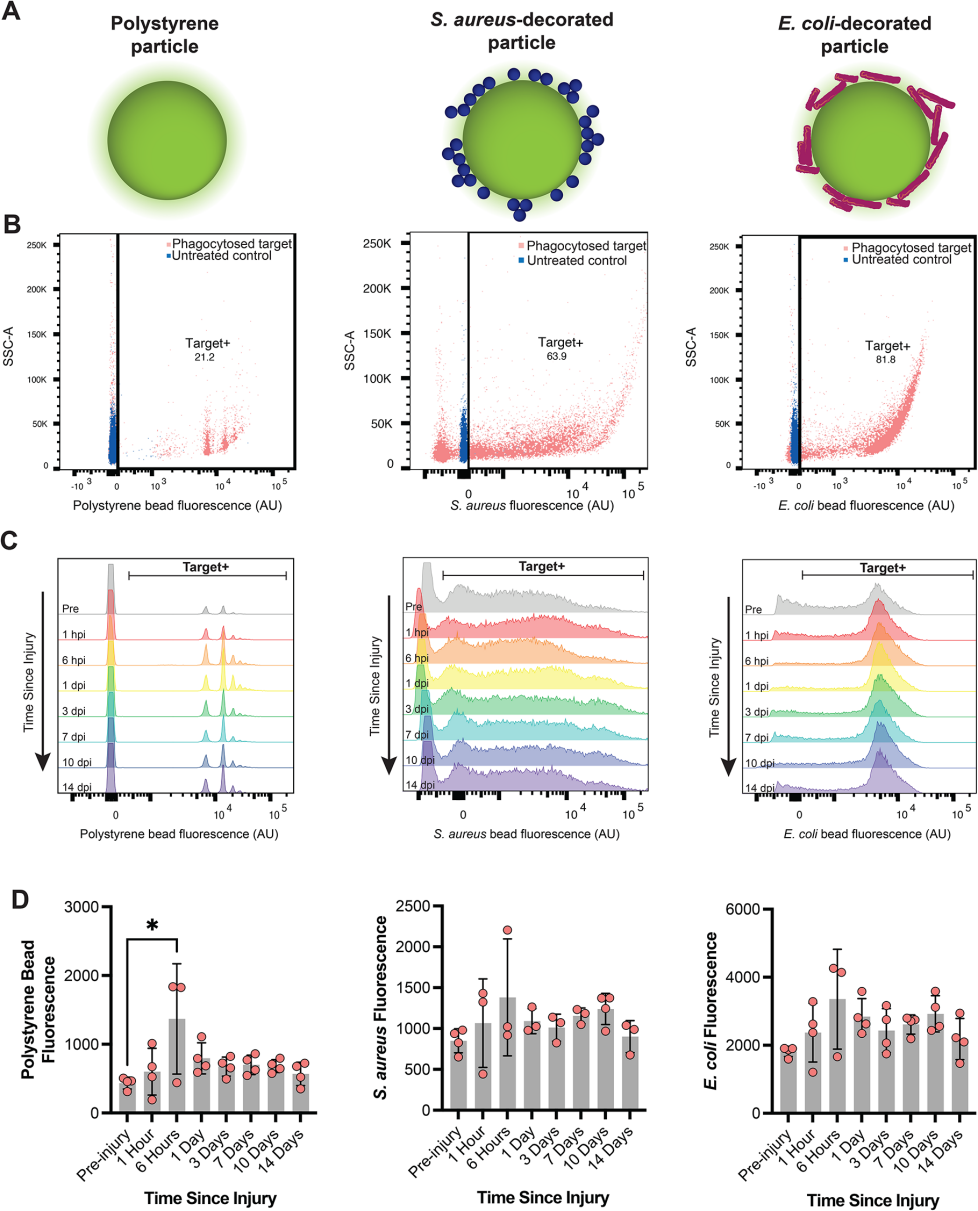
Changes to PBMC phagocytic uptake of different targets over time after a high rotational velocity TBI. Circulating PBMCs extracted over time after a high rotational velocity TBI were cultured with one of three targets: fluorescent polystyrene particles, fluorescent particles decorated with *S. aureus* fragments, or fluorescent particles decorated with *E. coli* fragments (**A**). Phagocytic uptake was observed across all targets (**B**) and timepoints (**C**). The total fluorescence of the extracted PBMC population over time (**D**)

Phagocytosis is another critical PBMC function, whereby immune cells identify and clear foreign contaminates, cells, or debris from the environment. To assess if phagocytic clearance was affected by TBI, extracted PBMCs were PMA-stimulated and then cultured with one of three targets: a fluorescent polystyrene particle, a fluorescent particle decorated with *S. aureus* fragments, or a fluorescent particle decorated with *E. coli* fragments (Fig. 6A). The polystyrene particle was used to mimic a foreign biomaterial, while the *S. aureus*-decorated particles and *E. coli*-decorated particles were used to mimic gram-positive and gram-negative microbial infections, respectively.

Culturing targets with extracted PBMCs resulted in phagocytic uptake in all cases (Fig. 6B-C). PBMCs extracted from animals experiencing a sham or a moderate rotational velocity TBI did not change the extent of phagocytosis over time across any of the targets (Supp. Figure 7). Interestingly, the extent of phagocytosis was altered against some targets in PBMCs extracted after a high rotational velocity TBI. Specifically, PMA-stimulated PBMCs extracted 6 hpi increased their phagocytic uptake of polystyrene particles relative to pre-injury levels (Fig. 6D). No significant changes in the extent of phagocytosis were observed when simulated PBMCs were cultured with *S. aureus*-decorated particles or *E. coli*-decorated particles. Increased uptake of polystyrene particles 6 hpi was driven by a significant increase in the number of target + cells, suggesting that this increased phagocytosis activity was driven by more PBMCs participating in clearing the foreign polystyrene particles (Supp. Figure 9). Together, these data suggest there is an injury severity, temporal, and target-specific response to phagocytosis clearance following brain injury.

### Plasma cytokine concentrations were unchanged following closed-head diffuse TBI

Cytokines are potent signaling molecules, which can be found in the plasma, and influence immunological homeostatic setpoints and responses with complex dynamics. The levels of circulating plasma cytokines in experimental rodent models of TBI exhibit variable trends across injury model, time, and severity [45, 46]. Likewise, cytokines in clinical cases of TBI exhibit high variability across patients and cohorts [47, 48]. Here, we assessed the concentration of ten major plasma cytokines across time and injury severity. Interleukin (IL)-1β, IL-6, IL-8, IL-12, interferon (IFN)-γ, granulocyte-macrophage colony-stimulating factor (GM-CSF), and tumor necrosis factor (TNF)-α are generally associated with mounting inflammation. IL-10, IL-4, and transforming growth factor (TGF)-β1 are associated with damped inflammation or non-classical immune activation. Across three injury conditions, all timepoints, and these 10 major cytokines, we observed no significant changes in the concentration of these plasma cytokines (Supp. Figure 10).

### Peripheral immune organ composition exhibits modest cellular changes following high rotational velocity TBI

**Fig. 7:**
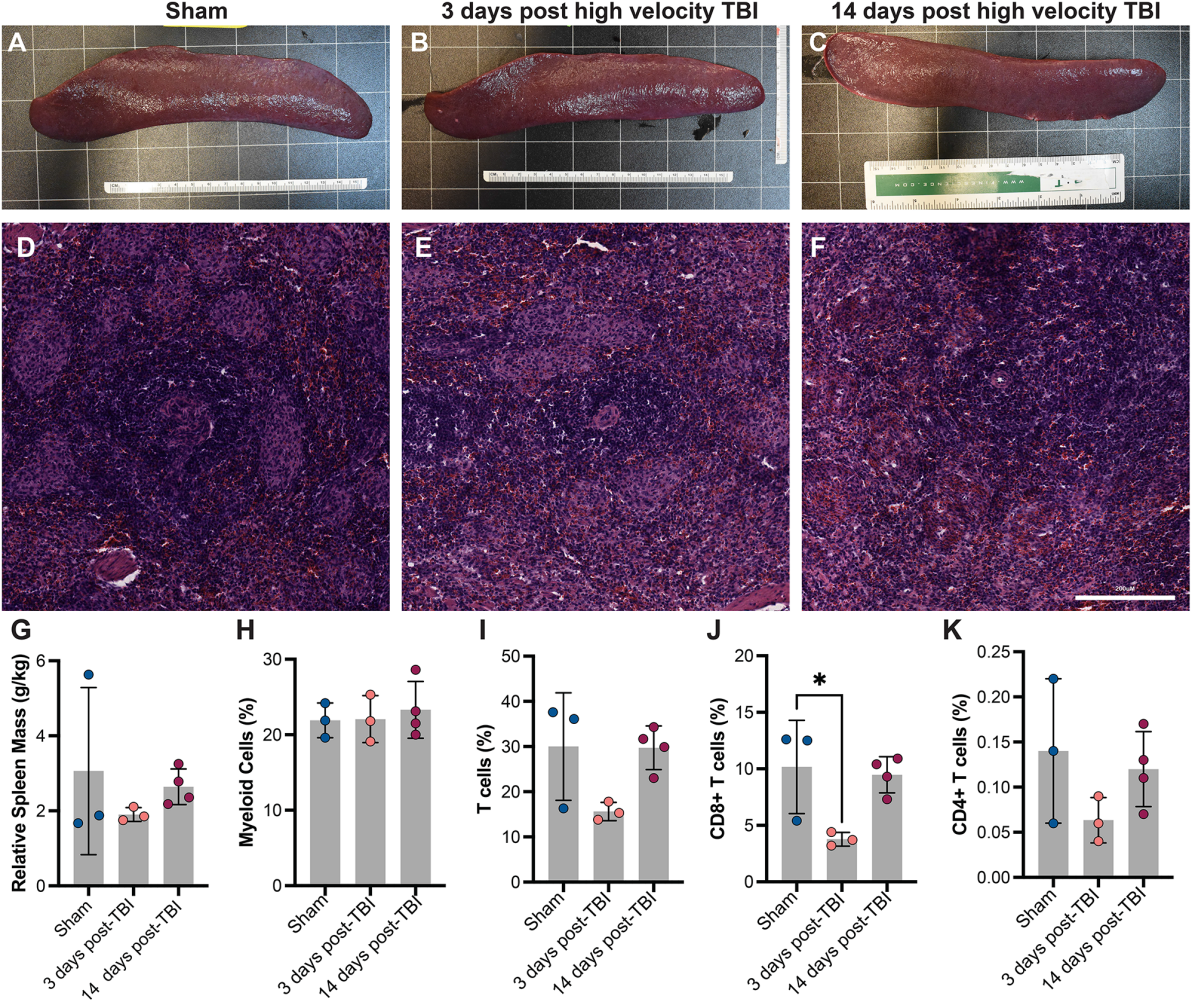
Changes to the spleen after a high rotational velocity TBI. Representative gross tissue images of the spleen after a sham injury (**A**) or a high rotational velocity injury (**B**-**C**). Representative H&E staining of splenic white and red pulp across injury condition and time (**D**–**F**). Relative spleen mass (**G**) and the percentage of myeloid and T cells does not change across condition. CD8^+^, but not CD4^+^ T cells, are significantly reduced 3dpi relative to sham levels (**J**-**K**)

**Fig. 8:**
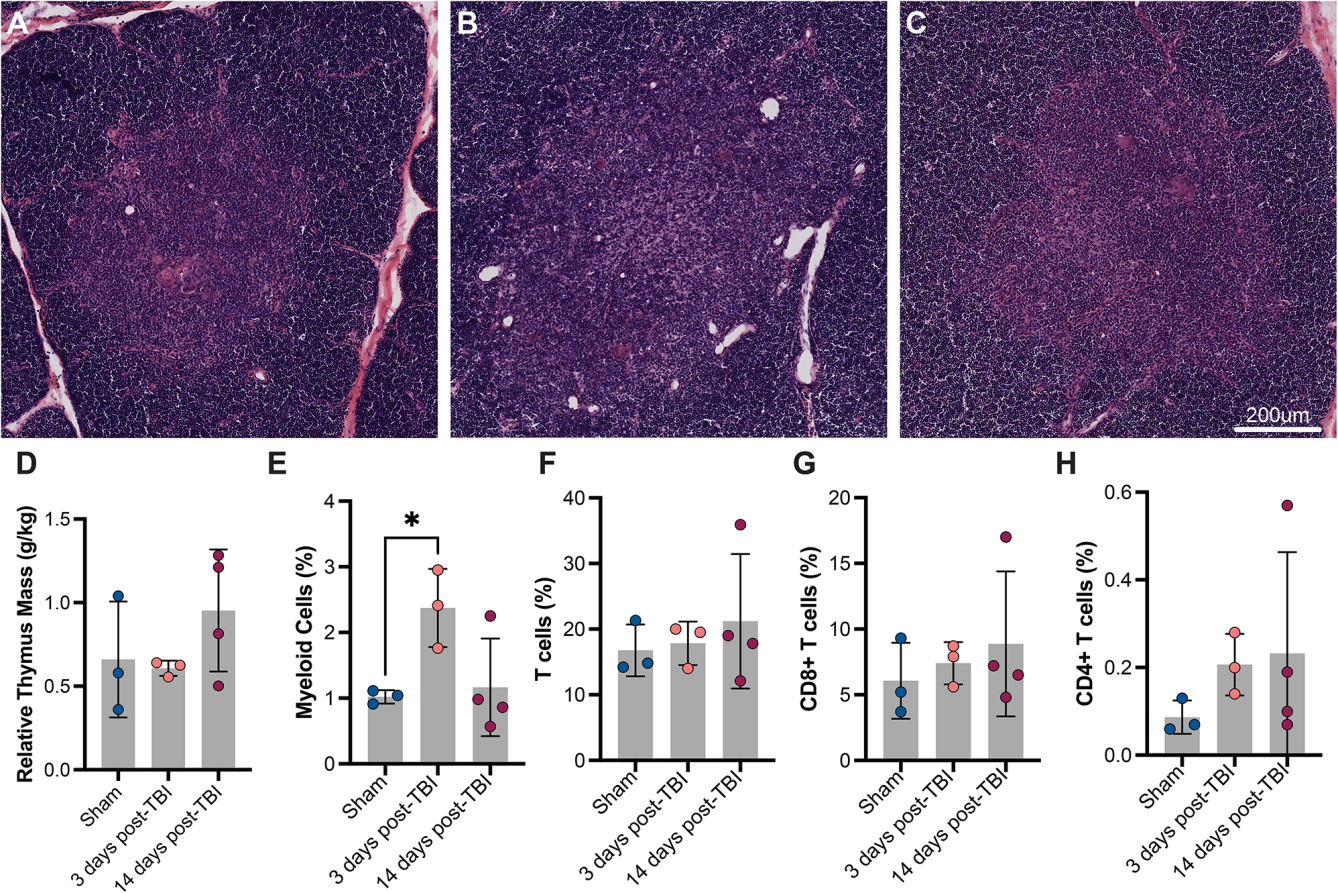
Changes to the thymus after a high velocity TBI. Representative H&E staining of thymus cortex and medulla after a sham injury or a high rotational velocity injury (**A**–**C**). Relative thymus mass (**D**) does not change across injury condition. The percentage of myeloid cells (**E**), but not T cells (**F**–**H**) increase 3dpi relative to sham levels

Following a high rotation velocity TBI or a moderate rotational velocity TBI, there were no significant changes to the spleen or thymus mass relative to sham levels (Figs. 7 and 8, Supp. Figure 11). A trained and blinded veterinary pathologist, scoring H&E-stained tissue, found no major differences in spleen or thymus tissue structure across injury conditions.

Fresh spleen tissue and thymus tissue were mechanically digested, stained, and assessed with flow cytometry to quantify splenocyte and thymocyte immune cell composition. In splenocytes, myeloid cells remained unchanged while T cells exhibited a non-significant decrease in population 3 dpi, which was driven by a decrease in the number of CD8 + cytotoxic T cells 3 dpi (Fig. 7H-J). CD4 + T cells comprised a small fraction of the splenocyte population, and they exhibited a non-significant decrease 3 dpi (Fig. 7K).

In thymocytes, the myeloid cell population significantly increased 3dpi relative to sham animals before returning to baseline levels at 14 dpi (Fig. 8E). T cells and their subsets remained unchanged in thymocytes 3 and 14 dpi (Fig. 8F-H). At 14 days post-injury, there were no significant changes to immune cell composition in splenocytes or thymocytes across sham, moderate rotational velocity, or high rotational velocity TBIs (Supp. Figure 11).

## Discussion

In this study, we assessed changes to the peripheral immune system following a closed-head diffuse TBI in pigs because the peripheral immune system regulates secondary infections and is a major contributor to neuroinflammation in the brain, neurological deficits, and neurodegeneration [8, 49]. We found that the peripheral immune system exhibits dysregulation of innate and adaptive cellular populations and their physiology following closed-head rotational TBI, with more injurious head rotational levels resulting in greater peripheral immune system dysregulation. Furthermore, we observed that the vast majority of cellular population, cellular function, and cytokine changes occurred within the first 10 days following a high rotational velocity TBI. These findings suggest that even following a closed-head diffuse TBI that does not generate major brain hemorrhage, cavitation, or lesions, there are changes to the peripheral immune system that persist out to subacute times post-injury.

In line with these findings, previous reports from clinical cases of severe TBI patients found the peripheral immune system is dysregulated following TBI [2, 3, 50]. TBI induces systemic inflammation acutely following severe trauma [50, 51]. Thereafter, humoral and neurogenic compensatory mechanisms can mount an anti-inflammatory response, leaving the body vulnerable to secondary infections and may stall endogenous tissue regeneration capabilities [2]. In support of this premise, severe TBI patients are more likely to experience nosocomial infections, sepsis, ventilator associated pneumonia, and death as a result of immunosuppression [2, 52, 53].

While our experimental model does not mimic key aspects of clinical severe TBI patients in intensive care units including chronic sedation, polytrauma, or administration of immunomodulatory medications, our findings mirrored several important clinical trends of severe TBI. In agreement with our data, clinical severe TBI and contusive TBI in mice has been associated with a transient increase in myeloid cells [3, 53–55]. Additionally, echoing clinical trends, we observed a decrease in the fraction of NK cells within PBMCs over time [53, 56].

Previous characterization of clinical TBI cases has also reported changes to circulating T cell populations [53, 57, 58]. After severe TBI, T cell numbers are significantly downregulated at acute timepoints before returning to the level of heathy controls [53, 57, 58]. In our pig model, we observed a similar, albeit non-significant, decrease in T cell concentration 6 h post-injury. Thereafter, T cell population numbers rebounded at later, subacute timepoints. The upregulation of circulating T cells at subacute timepoints was driven by increases to all T cell subpopulations. CD4 + T helper cells, which can differentiate into Th1, Th2, Th17, or T regulatory cells upon stimulation, can drive diverse functions including secreting pro- or anti-inflammatory cytokines, regulating other immune cells, inducing tissue homing, and affecting neuroinflammation [59]. CD8 + T cells respond to specific antigens, massively upregulating their populations in response to an immune challenge. Both CD4 + and CD8 + T cells play a role in TBI as well as neurodegeneration [49, 60]. The increase in immune cells at later timepoints may be partially driven by the decrease in splenic T cells 3 dpi, where exiting splenic T cells could be entering into the circulation.

The majority of the changes reported here occurred within the first 10 days following an injury. Indeed, the concentration of PBMCs, myeloid cells, T cells, and NK cells peaked 10 days post injury. Multiple immunological changes converging at this timepoint suggests that some secondary injury cascade, alteration in neurophysiology, peripheral signaling cascade, or tissue remodeling occurred well into the subacute timeframe in this injury model. These findings invite future work investigating the neurological, pathological, and immunological significance of this timepoint. Indeed, investigating infection susceptibility as well as the consequences of a secondary TBI at this timepoint could be especially informative given that secondary infection is a major driver of morbidity and mortality in clinical severe TBI [15, 61, 62].

While many of our findings mirror cellular changes in the clinical literature, deeper immunophenotyping is required to understand or infer consequences to immune system functionality. For example, subtyping myeloid cells into monocyte classes, determining HLA-DR expression, subtyping T cells, determining protein production, investigating crosstalk between immune cell populations, and completing immune challenges in vivo are necessary to fully interpret how changes in immune cell populations affect the body’s ability to fight foreign pathogens. Furthermore, characterization of immune cells procedurally excluded from these analyses, including neutrophils and granulocytes, will yield additional insights into changes to the peripheral immune system.

In addition to deeper immunophenotyping, this research invites investigations into how the altered peripheral immune system affects the damaged brain following TBI. In spite of limited hemorrhage in these animals, our data suggests that the BBB is highly permeable acutely following a high rotational velocity TBI. Previous work has also reported BBB permeability following a milder closed-head diffuse TBI in pigs [63]. Future work should investigate how the dysregulated peripheral immune system affects the injured central nervous system following TBI using this preclinical model.

As is the case for most large animal research, this work utilized a small number of animals in each experimental group. While we were able to detect significant changes over time by repeated measures analyses, we likely underreport the number of significant changes that could have been detected with a more fully powered cohort. For example, the concentration of T cells in circulation consistently exhibited a decreasing trend 6 h post injury across several metrics, although this trend was not significant. Consequently, a lack of statistical significance does not imply statistical equivalence. In addition to these enrollment and power limitations, reagents available for pig cell analyses are vastly more limited relative to mouse, rat, or human reagents. Generating a panel of flow cytometry antibodies for porcine immunophenotyping is quite challenging and has only been made possible recently. Further development of commercially available, pig-specific antibodies, reagents, and tools will facilitate the next phase of research in this injury paradigm and facilitate deeper immune phenotyping and characterization.

Phagocytosis, the uptake of foreign cells and debris by immune cells, has been reported to be affected in peripheral immune cells following severe TBI. Here, we observed an increase in phagocytic uptake of polystyrene particles six hours after a high rotational velocity TBI. This increase in phagocytic uptake of some but not all targets indicates that immune cell physiology may be altered in a target-specific manner at these timepoints. Interestingly, these data conflict with clinical reports and human immunological studies. *S. aureus* is an opportunistic pathogen and its presence is a risk factor for ventilator-associated pneumonia in TBI patients [2, 64]. *S. aureus* is the most common pathogen for hospital-acquired pneumonia, a major risk factor for hospitalized TBI patients [64, 65], insinuating that the immune response to this particular pathogen in TBI patients is muted or ineffective. Indeed, a previous report collected PBMCs from brain injury patients that presented with abnormal CT scans and systemic inflammatory response syndrome (SIRS), indicating a moderate-to-severe brain injury with immunosuppression. When these extracted PBMCs were cultured with fluorescent *S. aureus* bacteria, the percentage of phagocytic monocytes was decreased relative to healthy controls and remained low for at least six months [66]. Differences between our findings and clinical reports may be due to differences in species or due to the injury severity. Indeed, animal subjects exhibited minimal gross pathological changes and were completely ambulatory within a couple hours of the procedure, while clinical patients may be hospitalized and/or sedated for extended time periods and are typically intubated throughout their ICU stay. However, phagocytosis of pathogens is only one step in mounting a successful immune response. Examining antigen presentation, proliferation rates, and killing efficacy may also be perturbed in immune cells in response to this microbe.

These detectable changes to the immune system invite opportunities for biomarker discovery experiments. A major goal of the brain injury field has been to identify diagnostic and prognostic biomarkers for TBI. Using blood-based biomarkers has captured the attention of many research efforts because assaying the blood would allow for minimally invasive, repeated measures from injured patients. The majority of efforts have been focused on assessing proteins or brain byproducts from peripheral blood serum or plasma [67–70]. To date, using serum biomarkers to diagnose TBI severity or predict outcome has proven challenging because of the large variability of serum components across patients, requiring most studies to enroll hundreds of patients [67–69, 71–73]. When considering plasma cytokine concentrations over time, we were unable to detect significant differences across injury conditions or time for ten major immunomodulatory cytokines. However, despite subject variability, we were able to detect significant changes to immune cell populations with this modestly powered cohort. These data invite the opportunity for peripheral immune cells themselves, which are highly responsive to damage or disease, to serve as candidate diagnostic and prognostic biomarkers in isolation or combination with protein biomarkers [51, 74]. As previously mentioned, the immune system is a major sensor of damage, disease, or trauma and responds to these signals with rapid, robust, and occasionally protracted responses. Therefore, discriminating immune cell responses to TBI from responses to other stimuli such as peripheral trauma, disease, or infection, would be critical to implementing this practically and interpreting findings.

In addition to biomarker applications, understanding changes to the immune system invites opportunities for therapeutic interventions. This study does not identify the mechanisms through which brain injury affects peripheral immune functionality. It is possible that crosstalk through the hypothalamic-pituitary-adrenal axis, the sympathetic nervous system, and/or the parasympathetic nervous system mediate these observed effects. Acutely after a closed-head diffuse injury in pigs, cytokines were not affected, the concentration of lymphoid cells generally decreased, the proportion of circulating myeloid cells increased, and the extent of phagocytosis increased. At subacute timepoints, all cell populations measured here increased in concentration. These immune system shifts may align with sympathetic nervous system activation and catecholamine release, although relative spleen and thymus mass remained unchanged at later timepoints [2, 75, 76]. Future work further characterizing the specific mechanisms that affect baseline peripheral immune homeostasis and functionality are necessary to identify potential treatment targets for tailored therapeutic interventions.

These data suggest that even following a brain injury that does not generate massive hemorrhage, contusions, or lesions, the peripheral immune system exhibits altered cellular dynamics and physiology up to two weeks following injury. These findings invite future studies that could investigate immune changes across biological sex, age, more chronic timepoints, and the mechanism by which the damaged brain affects peripheral immunity. Indeed, biological sex affects brain injury, neuroinflammation, and immune system physiology [77–79]. Because this research was completed entirely in female pigs, future work should seek to determine if trends are conserved in male subjects.

TBI can disrupt balanced crosstalk of the innate and adaptive immune systems through changes to the hypothalamic-pituitary-adrenal axis, the sympathetic-adrenal-medullary axis, the parasympathetic nervous system, or through release of DAMPs and cytokines into the peripheral blood stream [2, 80–82]. Damage to specific neuronal populations, network connectivity, or responsiveness of the immune system prevents a robust, resolvable, and effective inflammatory response to foreign pathogens. A dysregulated immune response leaves the body vulnerable to infection and with diminished tissue regeneration capabilities. Furthermore, immunodysregulation and infection can contribute to exacerbated neuronal damage, delayed or incomplete functional recovery, and death. Understanding the mechanisms by which brain trauma affects immune system physiology could be used to inform therapeutic interventions aimed to bolster peripheral immune functionality [3, 51]. Finally, future investigations are required to determine how the disrupted peripheral immune system contributes to neuropathology or neurophysiological changes after brain injury [83]. Understanding how the disrupted peripheral immune system affects immune cell homing to tissues, affects signaling to the brain, and susceptibility to subsequent injuries are critical to understanding the interactions between the damaged brain and the peripheral immune system.

## Conclusion

The peripheral immune system is exquisitely sensitive to changes to tissues throughout the body. Here, we utilized a clinically-relevant model of closed-head diffuse TBI in pigs to characterize changes to the peripheral immune system across injury severities and time. We report changes to immune cell populations and physiology are perturbed at acute and subacute timepoints with disruptions exhibiting an injury-severity-dependent pattern. Together, these data link isolated brain injury to detectable changes in the periphery in a large animal preclinical model. Future studies further characterizing cellular changes and investigating the impact of these alterations on neurological pathological progression are critical to contextualizing these dynamics and their implications for clinical patients.

## Electronic supplementary material

Below is the link to the electronic supplementary material.

Supplementary Material 1: Supp. Figure 1. Flow cytometry gating strategy to characterize PBMCs. Samples were gated to exclude debris (A), doublets (B), and dead cells (C). Live cells were then gated on CD52 and CD172 to segregate myeloid and lymphoid cell populations (D). Lymphoid cells were separated into CD3 + T cells or CD3- NK cells (E). CD3 + T cells were gated for CD4a and CD8a signals (F). Blue, red, and orange populations are FMO or unstained controls. Supp. Figure 2. Changes to circulating immune cell demographics over time after a sham or a moderate rotational velocity TBI. Representative H&E images after a sham (A) or moderate rotational velocity TBI (F). The circulating PBMC concentration (B, G), myeloid cell fraction (C, H), T cell fraction (D, I), and NK cell fraction (E, J) after a sham or a moderate rotational velocity TBI. Supp. Figure 3. Percent change in circulating myeloid, T, and NK cells. Circulating PBMCs were collected after a high rotational velocity TBI (A), moderate rotational velocity TBI (B), or a sham procedure (C). Repeated measures subtracted pre-injury baseline levels to reduce animal-to-animal variability in myeloid cells, T cells, and NK cell populations. Supp. Figure 4. T cell subtype dynamics over time after a sham or a moderate rotational velocity TBI. T cell subsets over time after a sham (A) or moderate rotational velocity TBI (F). The percentage of circulating CD8^+^ (B, G), CD4^+^ (C, H), double positive (CD8^+^/CD4^+^; D, I), and double negative (CD8^−^/CD4^−^; E, J) T cell subset after a sham or a moderate rotational velocity TBI. Supp. Figure 5. The percentage of T cell subtypes within T cells. The percentage of circulating CD8^+^, CD4^+^, double positive (CD8^+^/CD4^+^), and double negative (CD8^−^/CD4^−^) cells within the CD3 + T cells after a high rotational velocity TBI (A), moderate rotational velocity TBI (B), or sham procedure (C). Supp. Figure 6. CD8 to CD4 T cell ratios. The ratio of CD8 to CD4 T cells over time across sham (A), moderate rotational velocity TBI (B), or high rotational velocity TBI (C). Supp. Figure 7. ROS production from stimulated PBMCs. ROS production from stimulated PBMCs that were extracted over time after a sham (A), moderate rotational velocity TBI (B), or high rotational velocity TBI (C). Supp. Figure 8. Phagocytosis changes over time. Changes in phagocytosis uptake across targets (A) of extracted PBMCs after a sham procedure (B) or a moderate rotational velocity TBI (C). Supp. Figure 9. Target-loaded PBMC population changes over time. Polystyrene particles (A) were phagocytosed by PBMCs at all timepoints (B). The number of bead + cells (C) and the fluorescence of the bead + cell population (D) trends after a high rotational velocity TBI. Supp. Figure 10. Changes to cytokines over time. Plasma cytokine levels over time following a sham, moderate rotational velocity TBI, or high rotational velocity TBI. Supp. Figure 11. Changes to spleen and thymus composition across injuries. Outcome measures for the spleen (A-F) and the thymus (G-L) were not significantly different across injury velocity. Sham and high rotational velocity TBI data is repeated from Figs. 7 and 8 to provide context for moderate rotational velocity TBI outcomes.

## Data Availability

The data reported in this study are available from the corresponding author upon reasonable request.

## Abbreviations

ARRIVE: Animal Research: Reporting of In Vivo Experiments
cNST: Caudal nucleus of the solitary tract
DAMPs: Damage-associated molecular patterns
DHR: Dihydrorhodamine
dpi: Days post-injury
EDTA: Ethylenediaminetetraacetic acid
FMO: Fluorescence minus one
GM-CSF: Granulocyte-macrophage colony-stimulating factor
hpi: Hours post-injury
H&E: Hematoxylin and eosin
IFN: Interferon
IL: Interleukin
PBMCs: Peripheral blood mononuclear cells
PMA: Phorbol 12-myristate 13-acetate
rad/s: Radians per second
ROS: Reactive oxygen species
SIRS: Systemic inflammatory response syndrome
Supp: Supplemental
TBI: Traumatic brain injury
TGF: Transforming growth factor
TNF: Tumor necrosis factor
