# Supplementary material for: Peripheral immune cell dysregulation following diffuse traumatic brain injury in pigs
Source: J Neuroinflammation. 2024 Dec 18;21:324. doi: 10.1186/s12974-024-03317-y (PMC11657926; doi:10.1186/s12974-024-03317-y)

Supp. Figure 1

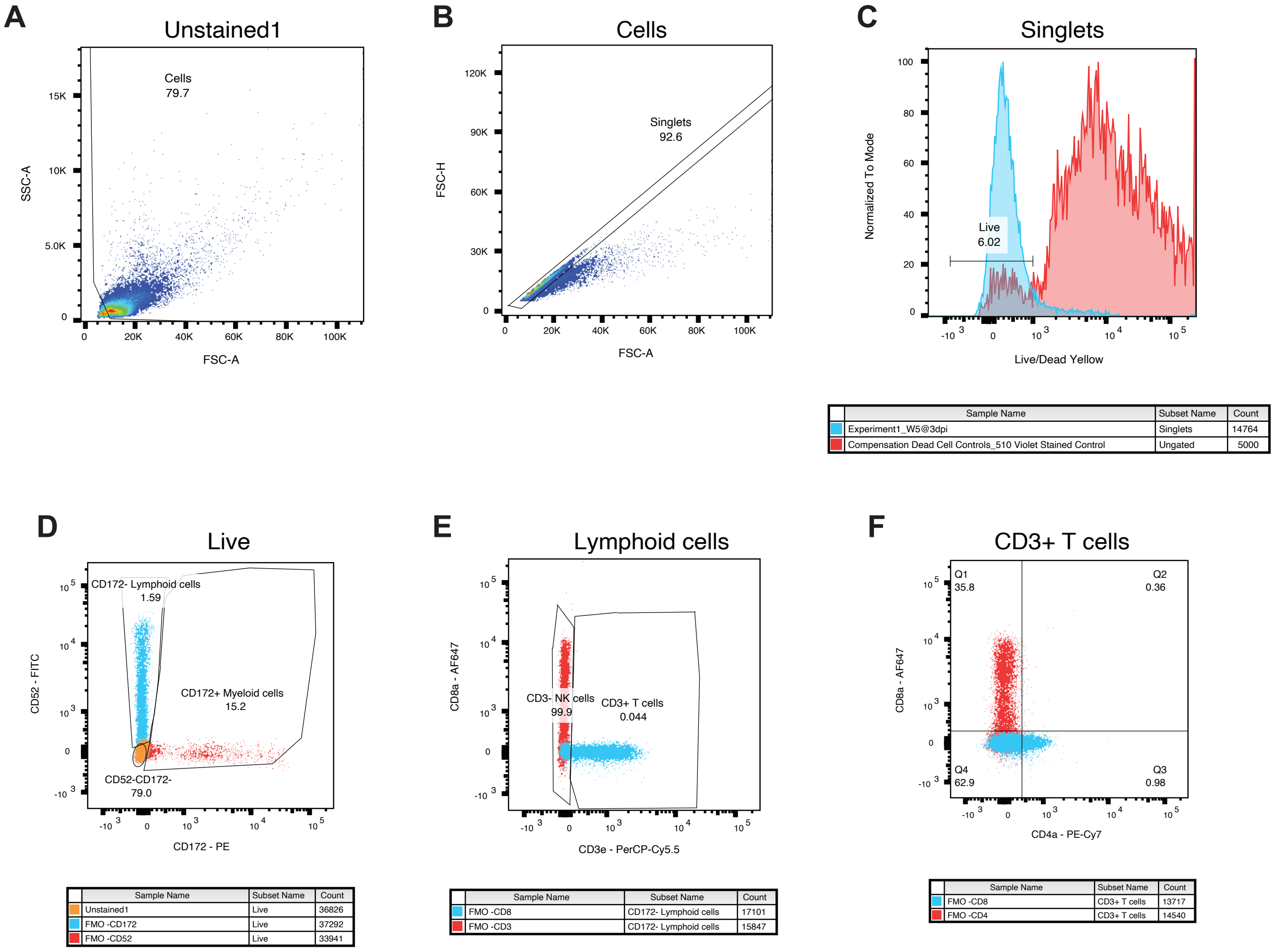

Supp. Figure 2

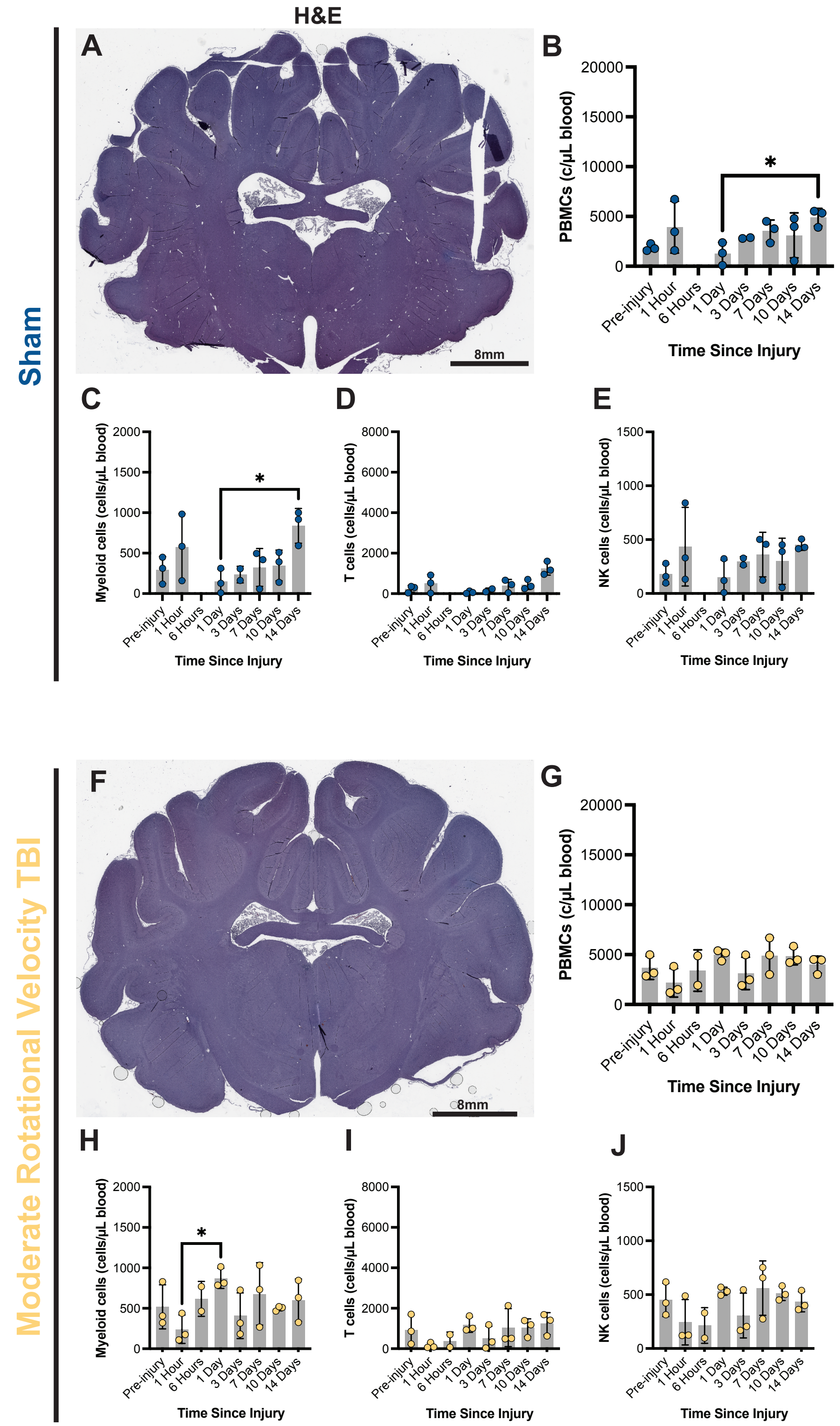

Supp. Figure 3

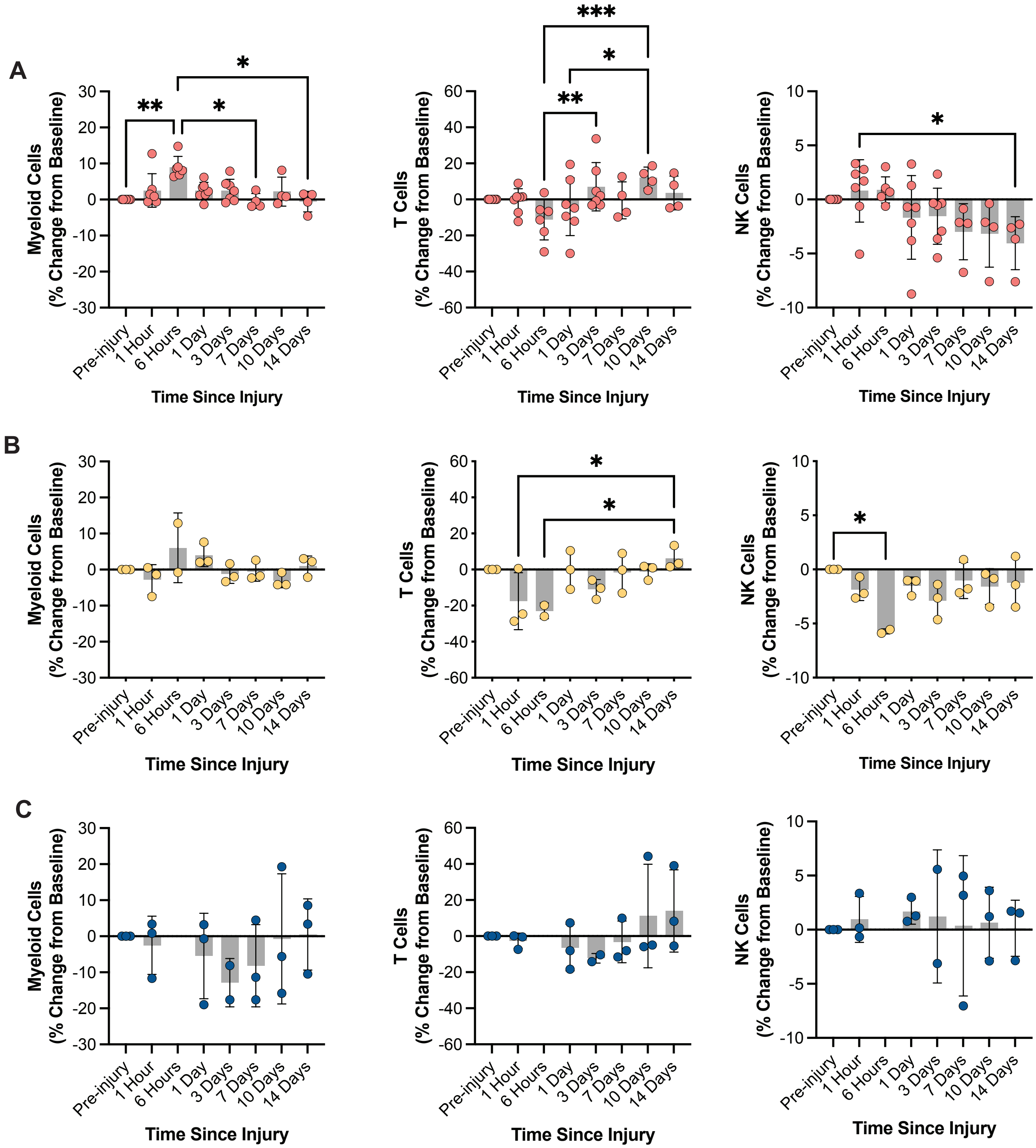

Supp. Figure 4

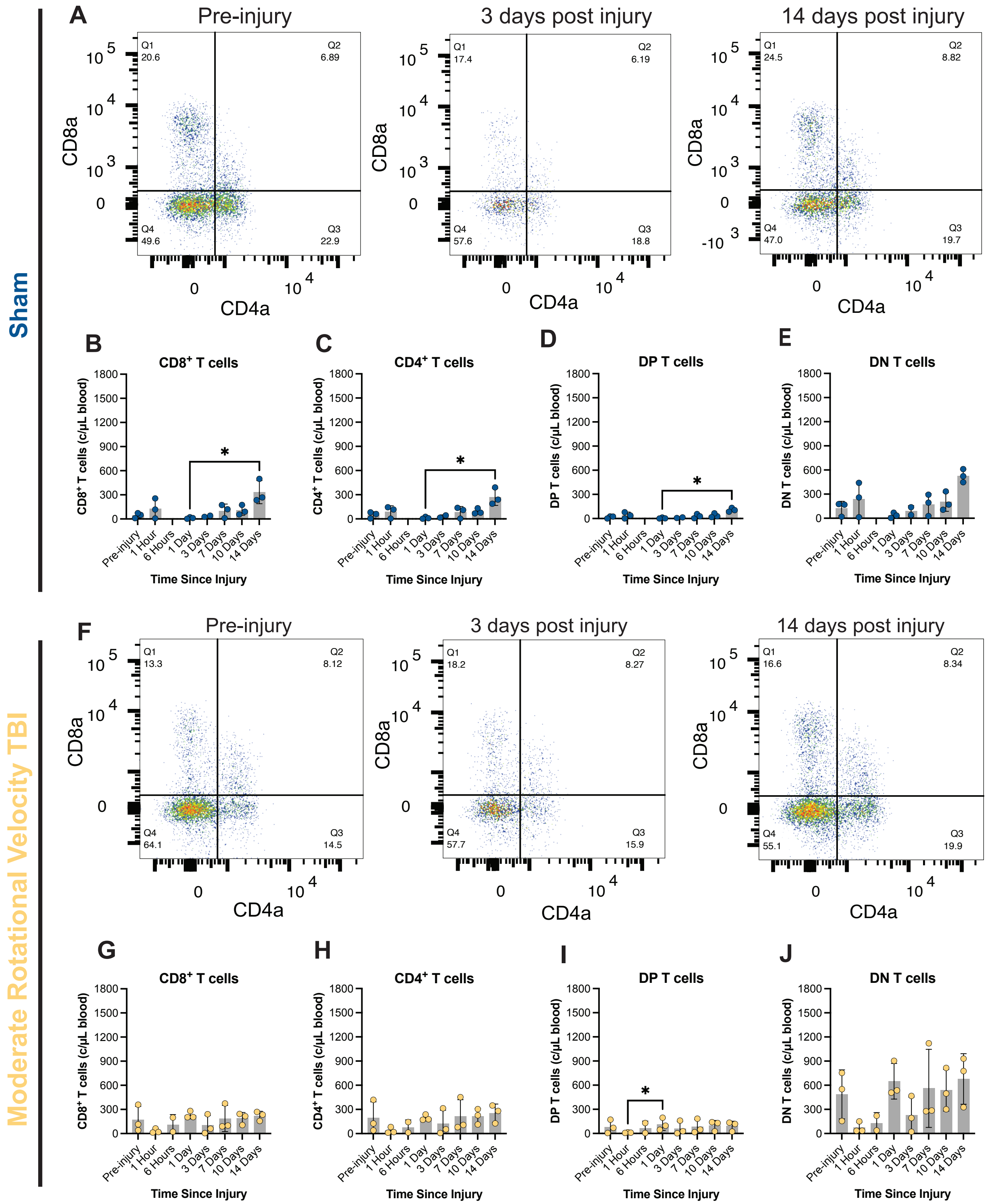

Supp. Figure 5

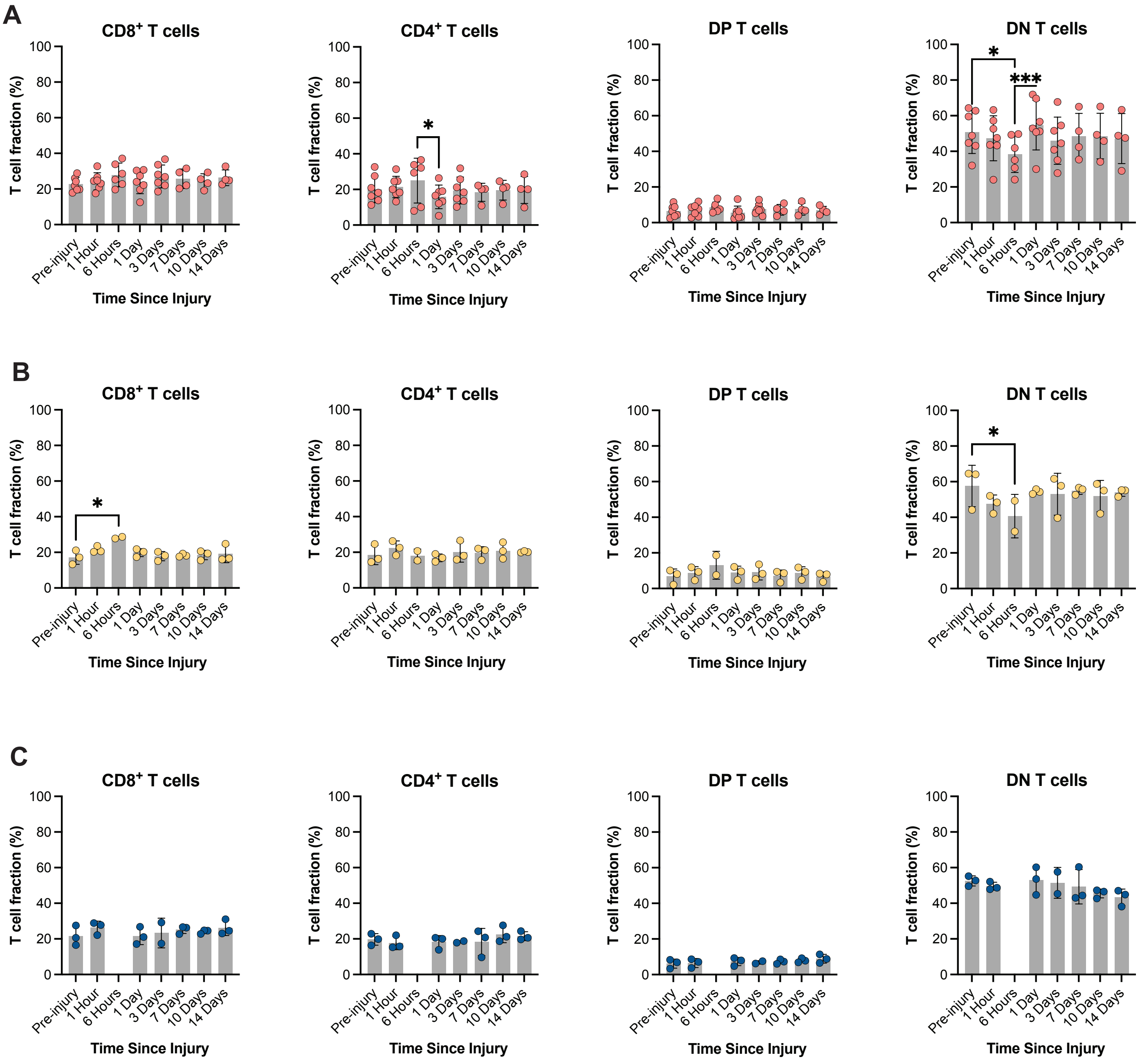

Supp. Figure 6

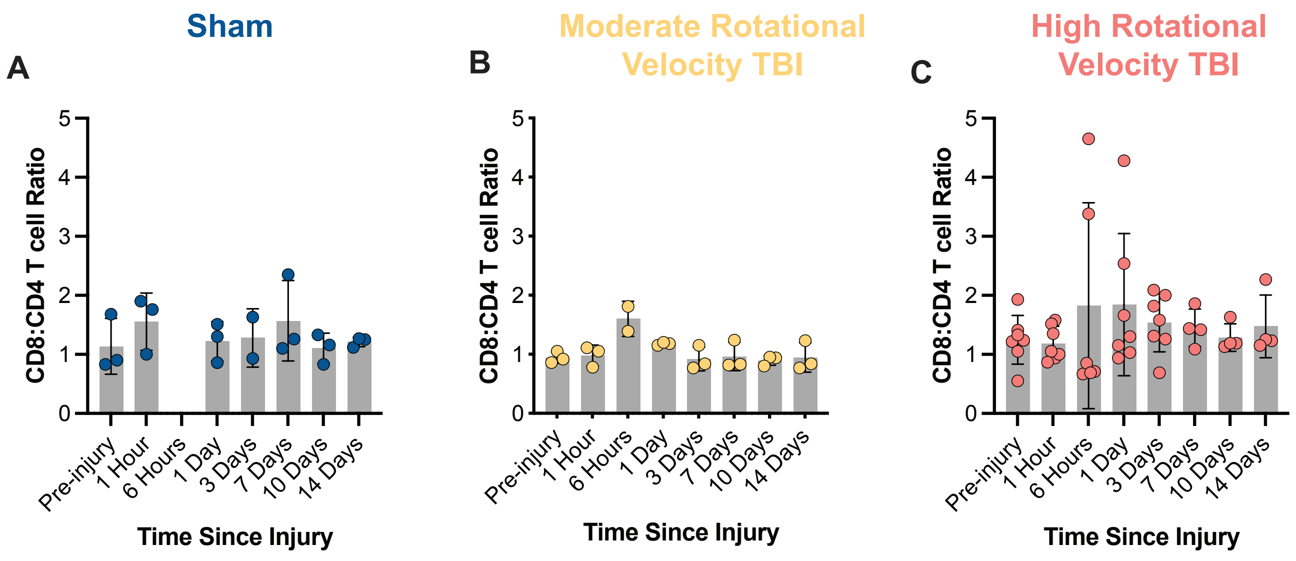

Supp. Figure 7

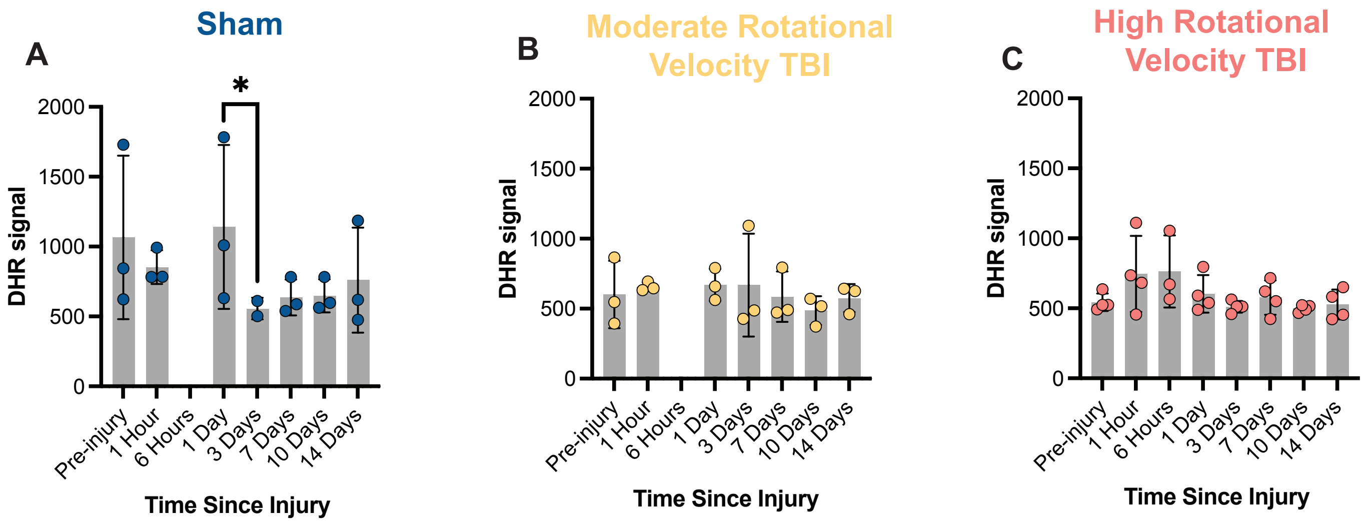

Supp. Figure 8

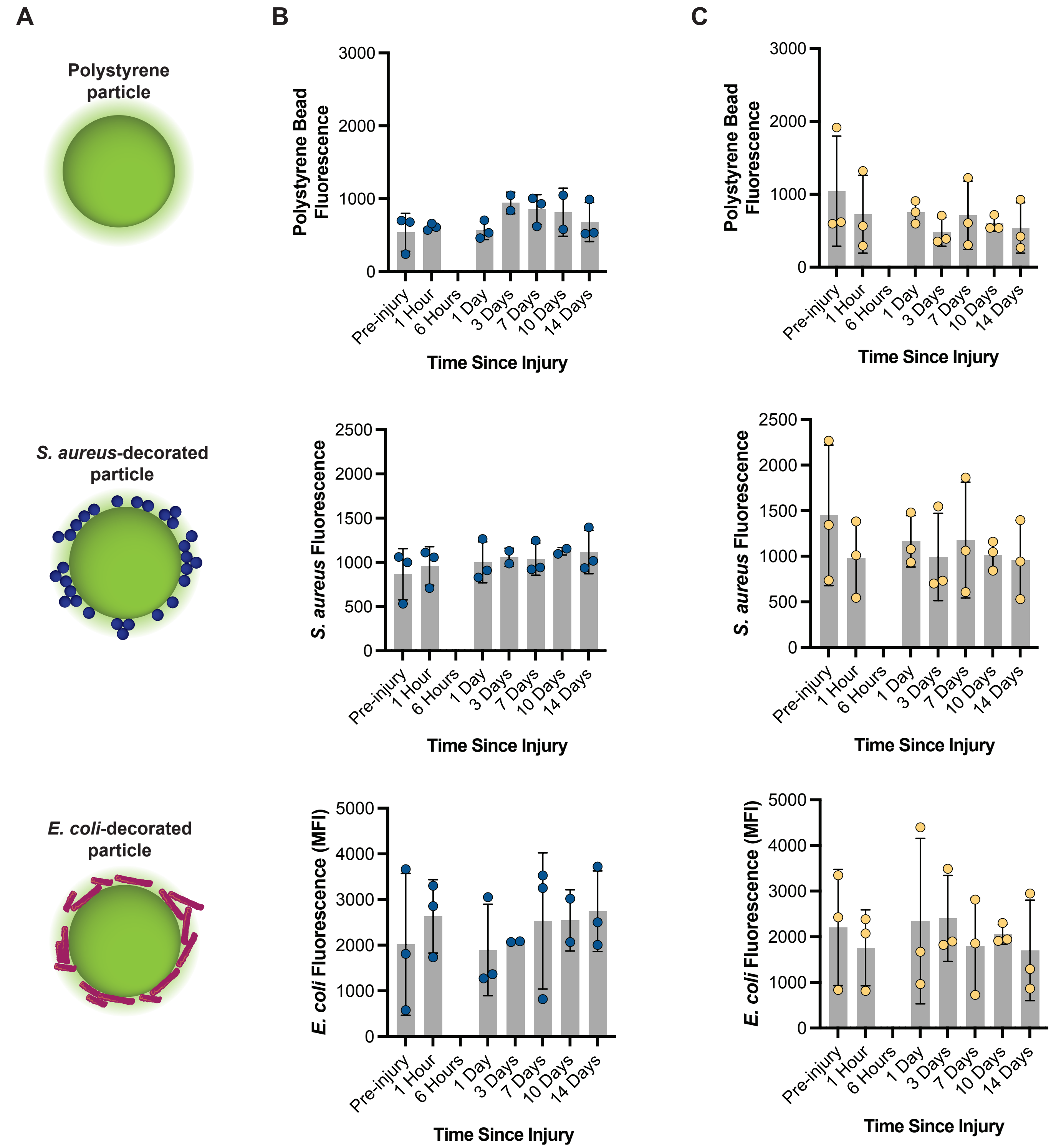

Supp. Figure 9

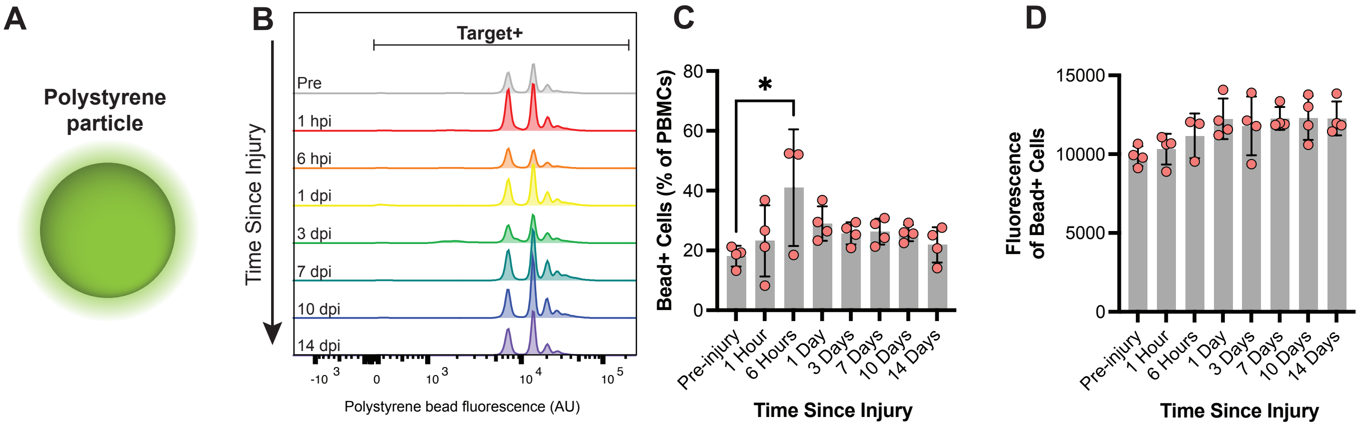

Supp. Figure 10

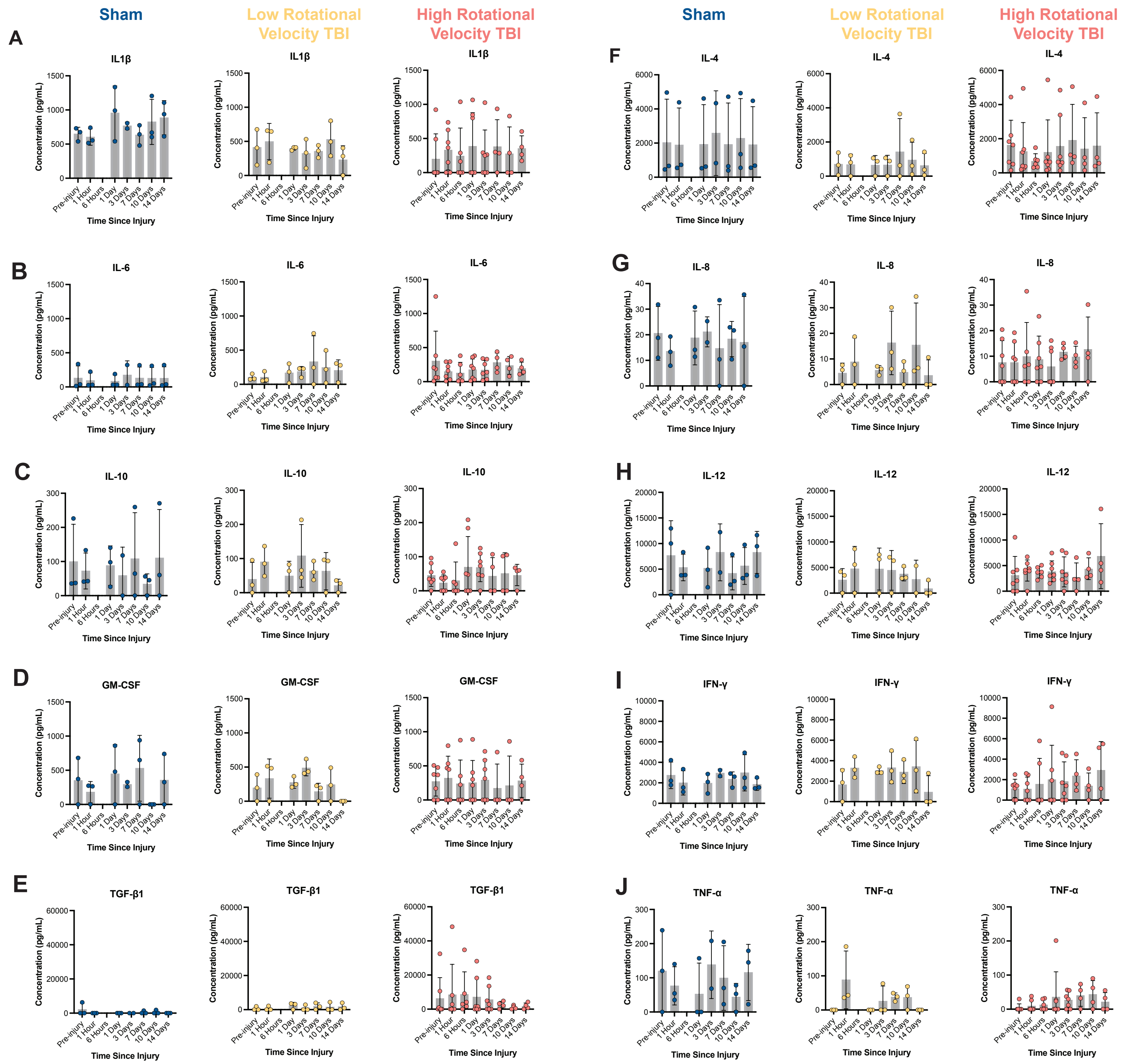

Supp. Figure 11

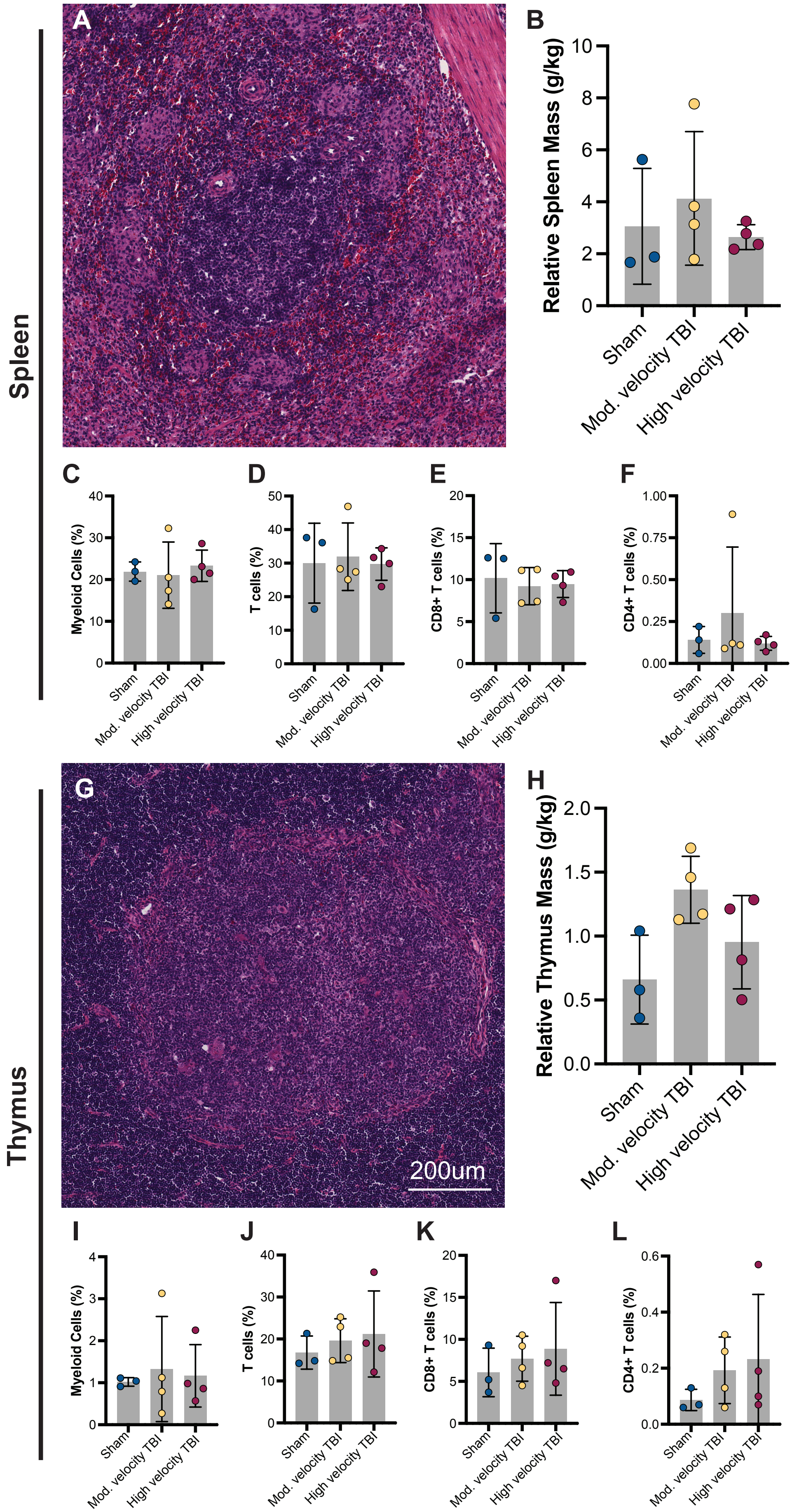

Supplement: Supplementary file 1 — Supplementary Material 1: Supp. Figure 1. Flow cytometry gating strategy to characterize PBMCs. Samples were gated to exclude debris (A), doublets (B), and dead cells (C). Live cells were then gated on CD52 and CD172 to segregate myeloid and lymphoid cell populations (D). Lymphoid cells were separated into CD3 + T cells or CD3- NK cells (E). CD3 + T cells were gated for CD4a and CD8a signals (F). Blue, red, and orange populations are FMO or unstained controls. Supp. Figure 2. Changes to circulating immune cell demographics over time after a sham or a moderate rotational velocity TBI. Representative H&E images after a sham (A) or moderate rotational velocity TBI (F). The circulating PBMC concentration (B, G), myeloid cell fraction (C, H), T cell fraction (D, I), and NK cell fraction (E, J) after a sham or a moderate rotational velocity TBI. Supp. Figure 3. Percent change in circulating myeloid, T, and NK cells. Circulating PBMCs were collected after a high rotational velocity TBI (A), moderate rotational velocity TBI (B), or a sham procedure (C). Repeated measures subtracted pre-injury baseline levels to reduce animal-to-animal variability in myeloid cells, T cells, and NK cell populations. Supp. Figure 4. T cell subtype dynamics over time after a sham or a moderate rotational velocity TBI. T cell subsets over time after a sham (A) or moderate rotational velocity TBI (F). The percentage of circulating CD8+ (B, G), CD4+ (C, H), double positive (CD8+/CD4+; D, I), and double negative (CD8−/CD4−; E, J) T cell subset after a sham or a moderate rotational velocity TBI. Supp. Figure 5. The percentage of T cell subtypes within T cells. The percentage of circulating CD8+, CD4+, double positive (CD8+/CD4+), and double negative (CD8−/CD4−) cells within the CD3 + T cells after a high rotational velocity TBI (A), moderate rotational velocity TBI (B), or sham procedure (C). Supp. Figure 6. CD8 to CD4 T cell ratios. The ratio of CD8 to CD4 T cells over time across sham (A), moderat [file 12974_2024_3317_MOESM1_ESM.pdf]
